# Major Structural Differences and Novel Potential Virulence Mechanisms from the Genomes of Multiple *Campylobacter* Species

**DOI:** 10.1371/journal.pbio.0030015

**Published:** 2005-01-04

**Authors:** Derrick E Fouts, Emmanuel F Mongodin, Robert E Mandrell, William G Miller, David A Rasko, Jacques Ravel, Lauren M Brinkac, Robert T DeBoy, Craig T Parker, Sean C Daugherty, Robert J Dodson, A. Scott Durkin, Ramana Madupu, Steven A Sullivan, Jyoti U Shetty, Mobolanle A Ayodeji, Alla Shvartsbeyn, Michael C Schatz, Jonathan H Badger, Claire M Fraser, Karen E Nelson

**Affiliations:** **1**The Institute for Genomic Research, RockvilleMarylandUnited States of America; **2**Produce Safety and Microbiology Research Unit, Agricultural Research ServiceUnited States Department of Agriculture, Albany, CaliforniaUnited States of America; Stanford UniversityUnited States of America

## Abstract

Sequencing and comparative genome analysis of four strains of *Campylobacter* including C. lari RM2100, C. upsaliensis RM3195, and C. coli RM2228 has revealed major structural differences that are associated with the insertion of phage- and plasmid-like genomic islands, as well as major variations in the lipooligosaccharide complex. Poly G tracts are longer, are greater in number, and show greater variability in C. upsaliensis than in the other species. Many genes involved in host colonization, including *racR/S, cadF, cdt, ciaB,* and flagellin genes, are conserved across the species, but variations that appear to be species specific are evident for a lipooligosaccharide locus, a capsular (extracellular) polysaccharide locus, and a novel *Campylobacter* putative *licABCD* virulence locus. The strains also vary in their metabolic profiles, as well as their resistance profiles to a range of antibiotics. It is evident that the newly identified hypothetical and conserved hypothetical proteins, as well as uncharacterized two-component regulatory systems and membrane proteins, may hold additional significant information on the major differences in virulence among the species, as well as the specificity of the strains for particular hosts.

## Abstract 2

Although the single genome sequence of the human *Campylobacter jejuni* yielded some insight, comparison of multiple species of the same genus elucidated greater understanding of virulence mechanisms

## Introduction

The Gram-negative, spiral-shaped bacterium Campylobacter jejuni is commensal in cattle, swine, and birds [1]. *Campylobacter* species, however, are the major cause of human bacterial gastroenteritis, and may be responsible for as many as 400–500 million cases worldwide each year [2]. Although the genus *Campylobacter* is composed of 16 described species [3], human illness is associated primarily with C. jejuni and C. coli and infrequently with *C. upsaliensis, C. lari,* and C. fetus. Filtration-based isolation techniques have revealed C. upsaliensis to be associated with human disease more than previously known [4]. The majority of C. jejuni infections result in uncomplicated gastroenteritis, but the development of the peripheral neuropathies, Guillain-Barré and Miller-Fisher syndromes is often associated with prior C. jejuni infection [5,6].

All clinically relevant *Campylobacter* spp. are considered to be thermotolerant in nature. *C. jejuni, C. coli, C. lari,* and C. upsaliensis also grow readily under microaerophilic conditions (5% oxygen) at 37 °C, and the majority of strains from these species will also grow at 42 °C. The thermotolerant *Campylobacter* spp. can also be distinguished by their host range. C. jejuni and C. coli are commensal in cattle, swine, and birds [1]; however, C. jejuni is often the predominant species in poultry, and C. coli in swine [4,7]. C. lari is prevalent in birds (seagulls in particular) [8], but has also been isolated from dogs and swine [9,10]. C. upsaliensis has frequently been isolated from domestic dogs and cats [11,12,13,14,15].

The main route of C. jejuni and C. coli human infection is through improperly handled or undercooked poultry, although illnesses caused by the consumption of livestock meat, unpasteurized milk, and contaminated water have also been reported [1]. C. lari has been isolated infrequently from poultry, ox and pork livers [16,17,18], and produce [19], in contrast to frequent isolation at moderate to high levels from fresh water, seawater, and shellfish [20,21]. C. upsaliensis has been isolated infrequently from poultry, ducks, and shellfish, and not from other food sources [4,22,23]. The main reservoir of C. upsaliensis appears to be dogs and cats, with reports of transmission of C. upsaliensis from animal to person [24,25] or person to person [26,27]. Human illness caused by C. lari and *C. upsaliensis,* unlike C. jejuni and *C. coli,* may be due to proximity to water and shellfish, and handling of pets, livestock, or livestock carcasses.

The genome sequence of C. jejuni strain NCTC 11168 [28], a human clinical isolate, provided a starting point for studying the proteins involved in outer surface structures and glycosylation [29], and the expression of contingency gene products such as glycosyl transferases and restriction enzymes. However, in contrast to the current understanding of the pathophysiology of other enteric bacteria, that of *Campylobacter* species remains poorly understood.

The genome of one C. jejuni strain is insufficient to provide a complete picture of the major aspects of *Campylobacter* biology, including the colonization of reservoir hosts [30], variation in lipooligosaccharide (LOS) and capsule, and potential adaptations of *Campylobacter* in poultry production and processing environments. In addition, information on the basis of *Campylobacter* virulence and potential targets for drug and vaccine design is still lacking. Therefore, we sequenced and finished the genome of C. jejuni strain RM1221 ( ATCC BAA-1062), and compared it with the genomes of C. coli strain RM2228 ( ATCC BAA-1061), C. lari strain RM2100 ( ATCC BAA-1060), and C. upsaliensis strain RM3195 ( ATCC BAA-1059) sequenced to at least 8-fold coverage. Strain RM1221 was sequenced because it was isolated from a chicken carcass and minimally passaged [31]. In addition, experimental work with this isolate has identified a number of unique features not present in the previously sequenced C. jejuni strain NCTC 11168, including the colonization of chicken skin and ceca, invasion of Caco-2 cells [31], unique LOS and capsule loci, and other unique open reading frames (ORFs) (unpublished data). C. coli RM2228 was sequenced because it is a multi-drug-resistant chicken isolate. Both C. lari RM2100 (CDC strain D67, “case 6” [32]) and C. upsaliensis RM3195 were selected for sequencing because they are clinical isolates. C. upsaliensis RM3195 was isolated from a patient with Guillain-Barré syndrome, using a filtration-based method of selection [33], and may have been responsible for this disease.

## Results/Discussion

### Comparative Genome Features

**Figure 1:**
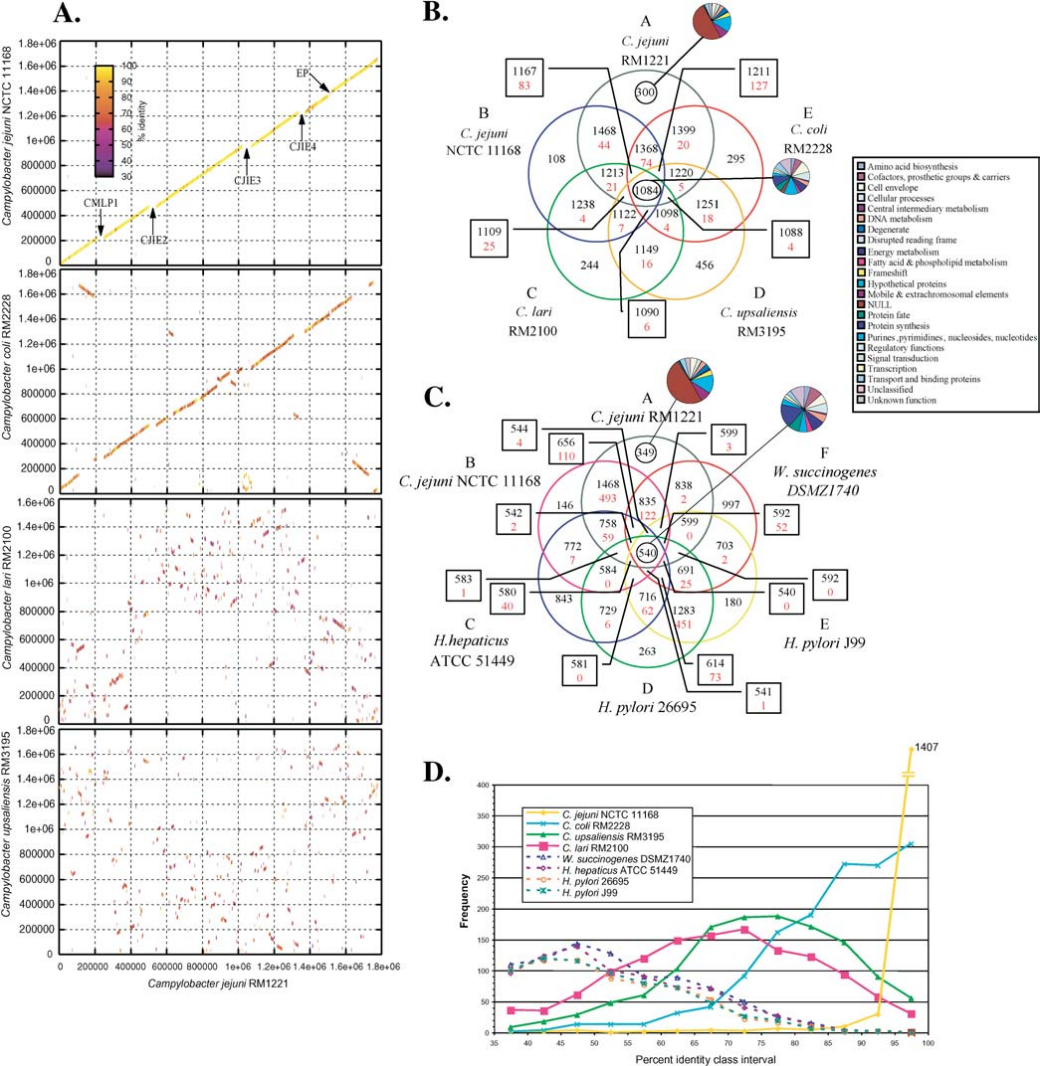
Whole-Genome Comparison of Five *Campylobacter* Strains Line figures depict the results of PROmer analysis. Colored lines denote percent identity of protein translations and are plotted according to the location in the reference (C. jejuni RM1221, x-axis) and query genomes (C. jejuni NCTC 11168 [upper y-axis] and C. coli RM2228 [lower y-axis]) (A). The Venn diagrams show the number of proteins shared (black) or unique (red) within a particular relationship for all five *Campylobacter* strains (B) and for members of the sequenced ɛ-Proteobacteria compared in this study (C). Protein sequences binned as “unique” are unique within the context of the genomes plotted and the cutoffs used to parse the BLASTP data. The pie charts plot the number of protein sequences by main functional role categories for C. jejuni RM1221 ORFs. A frequency distribution of protein percent identity (D) was computed: specifically, the number of protein sequences within class intervals of 5% amino acid identity from 35% to 100% that match C. jejuni RM1221 reference sequences were plotted.

The genome of C. jejuni RM1221 is a single circular chromosome, 1,777,831 bp in length, with an average G+C content of 30.31%. There are a total of 1,884 predicted coding regions in the genome with an average ORF length of 885 bp. Ninety-four percent of the genome represents coding sequence. Putative role assignments could be made for 1,124 of the ORFs (60%) (Table 1; Figure S1). The bacterium was found to belong to multilocus sequence type (MLST) 354 and FlaA short variable region (SVR) 33, which belongs to clonal complex 354, whose members are associated with human disease or chickens/chicken meat (Table 1) [34]. The genome features for the unfinished *Campylobacter* genomes were based on automated analysis and are presented in Table 1. The average coverage of the unfinished genomes was found to be 8.5-fold for C. coli RM2228, 16.5-fold for C. lari RM2100, and 9.0-fold for C. upsaliensis RM3195 for those contigs used to construct the pseudomolecules. The ambiguity rate (number of consensus-altering ambiguities per basepair) was determined to be between 1:54,000 and 1:93,000 for these unedited, unfinished genomes at 8-fold depth of coverage. The genomic structure of C. jejuni RM1221 is syntenic with the genome of C. jejuni NCTC 11168, and is disrupted by inserted prophages/genomic islands in RM1221 (see below), and ORFs within the capsular (extracellular) polysaccharide (EP) loci in NCTC 11168 (Figures 1A and S2). The C. coli RM2228 genomic structure also has a considerable amount of synteny with C. jejuni RM1221, sharing similar breakpoints, as observed in the C. jejuni comparisons, but displaying evidence of rearrangements about the oriC, as described for other bacterial genomes [35]. In contrast, C. lari and C. upsaliensis possess little if any synteny with C. jejuni RM1221.

**Table 1:**
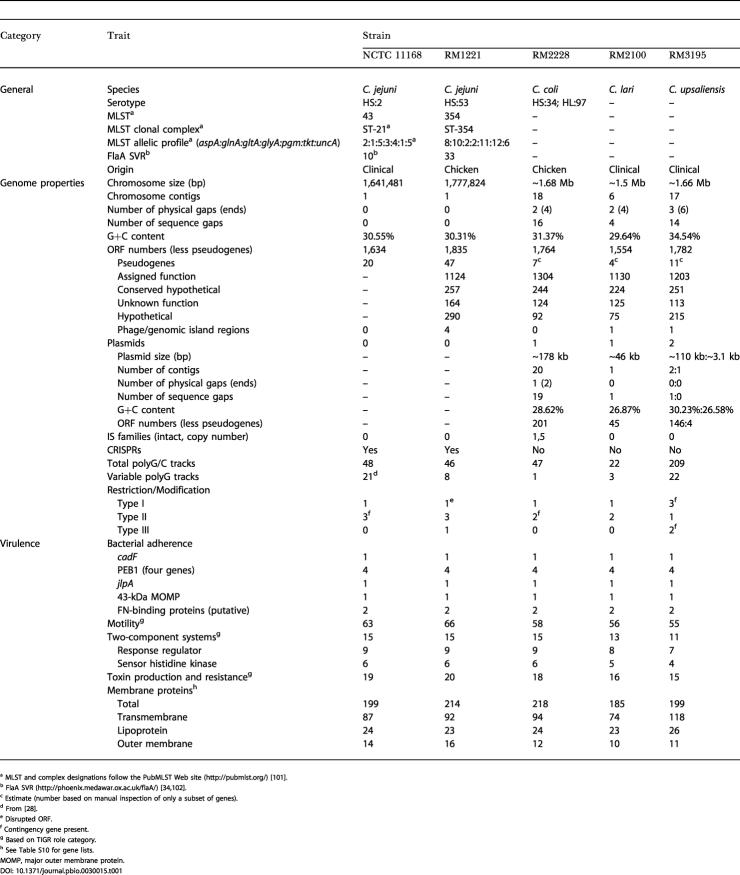
Genome Features of Five *Campylobacter* Genomes

^a^ MLST and complex designations follow the PubMLST Web site (http://pubmlst.org/) [101]

^b^ FlaA SVR (http://phoenix.medawar.ox.ac.uk/flaA/) [34,102]

^c^ Estimate (number based on manual inspection of only a subset of genes)

^d^ From [28]

^e^ Disrupted ORF

^f^ Contingency gene present

^g^ Based on TIGR role category

^h^ See Table S10 for gene lists

MOMP, major outer membrane protein

Comparison of C. jejuni RM1221 protein sequences with those of other fully sequenced members of the ɛ-Proteobacteria revealed 540 shared protein sequences, many of which are proposed to have “house-keeping” functions (Figure 1C). Of the 1084 protein sequences shared by all the *Campylobacter* species in this study, 46 had no match to any other organism in the database (*p*-value cutoff ≤ 10^−5^) (Figure 1B). Eleven of these were assigned functions related to cell envelope biosynthesis, or fatty acid and phospholipids metabolism. Further analysis revealed 44 proteins considered C. jejuni-specific, of which 12 mostly hypothetical proteins were truly novel, having no match to other organisms in the database. Of the 300 C. jejuni RM1221-specific protein sequences, only 95 were not in phage or genomic island regions.

To quantify relatedness among the sequenced ɛ-Proteobacteria, the average protein percent identity was computed for all proteins matching the reference strain C. jejuni RM1221 with a *p*-value less than or equal to 10^−5^, identity of 35% or more, and match lengths of at least 75% of the length of both query and subject sequence. Not surprisingly, C. jejuni NCTC 11168 had the highest average protein percent identity (1,468 proteins averaging 98.41% identity) with C. jejuni RM1221 proteins. C. coli RM2228 was second, with 1,399 proteins averaging 85.81% identity. Surprisingly, C. upsaliensis RM3195 had the third highest average protein percent identity with C. jejuni RM1221 (1,261 proteins; 74.72% average identity), followed by C. lari RM2100 with 1,251 proteins having 68.91% average identity. This was surprising since a 16S rRNA tree depicts C. upsaliensis to be more dissimilar to *C. jejuni, C. coli,* and C. lari [3]. Wollinela succinogenes DSMZ1740 was next, with 838 proteins averaging 53.77% identity, followed by Helicobacter hepaticus ATCC 51449 (770 proteins; 53.66% average identity), H. pylori 26695 (675 proteins; 52.39% average identity), and H. pylori J99 (682 proteins; 52.28% average identity).

### Phylogenetic Comparisons

**Figure 2:**
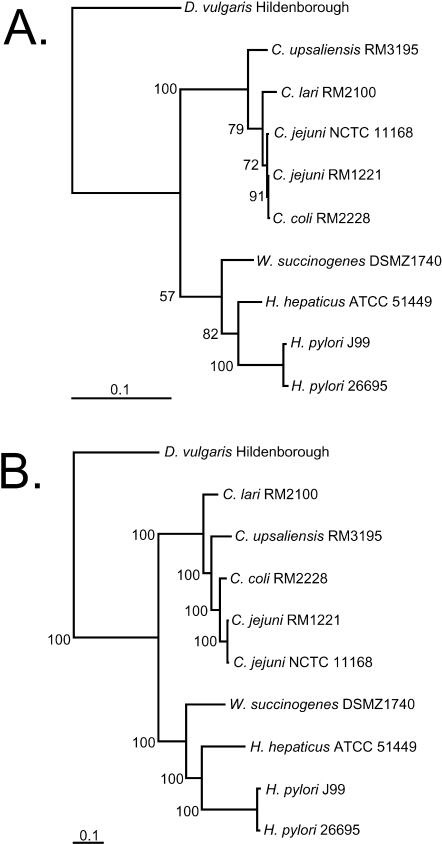
Phylogenetic Analysis and Frequency Distribution of Protein Percent Identity Concensus maximum-likelihood trees are depicted using multiple alignments of 16S rRNA (A) or 12 concatenated protein datasets (B). The numbers along the branches denote percent occurrence of nodes among 100 bootstrap replicates. The scale bar represents the number of nucleotide (A) or amino acid (B) substitutions.

To resolve the apparent discrepancy regarding the relatedness of the ɛ-Proteobacteria between the results of average protein percent identities from this study and the previously published 16S rRNA tree based on percent sequence similarity [3], a consensus boot-strapped maximum-likelihood tree was generated based on trimmed alignments with gaps removed (Figure 2A). One of the advantages of generating whole-genome sequence is the magnitude of information available for resolving differences between closely related organisms. To better resolve the *Campylobacter* species, we took advantage of the wealth of sequence information to construct a maximum-likelihood concatenated protein tree using a set of 12 conserved protein sequences that have been previously shown to be reliable markers for phylogenetic analysis (Figure 2B) [36,37]. A frequency distribution of protein percent identity was plotted with 5% class intervals to visualize the similarities of these genomes at the protein level (see Figure 1D). The 16S rRNA tree of sequenced members of the ɛ-Proteobacteria suggests that C. jejuni RM1221 is more closely related to C. coli RM2228 than to the other C. jejuni strain, NCTC 11168. However, the concatenated protein tree of these same organisms showed the two C. jejuni strains to be more closely related to each other than either is to C. coli RM2228, agreeing with the distributions of protein percent identities (see Figure 1D). Both trees indicate that W. succinogenes is more closely related to *Helicobacter* than to *Campylobacter*. Most likely, the protein tree is more accurate and the rRNA tree is incorrect because the 16S rRNA does not have enough variation to resolve these close relationships [37]. Whole-genome sequencing of more members of the ɛ-Proteobacteria will enable a clearer resolution of the evolutionary relationships within this group of related organisms.

### Phages/Genomic Islands

**Figure 3:**
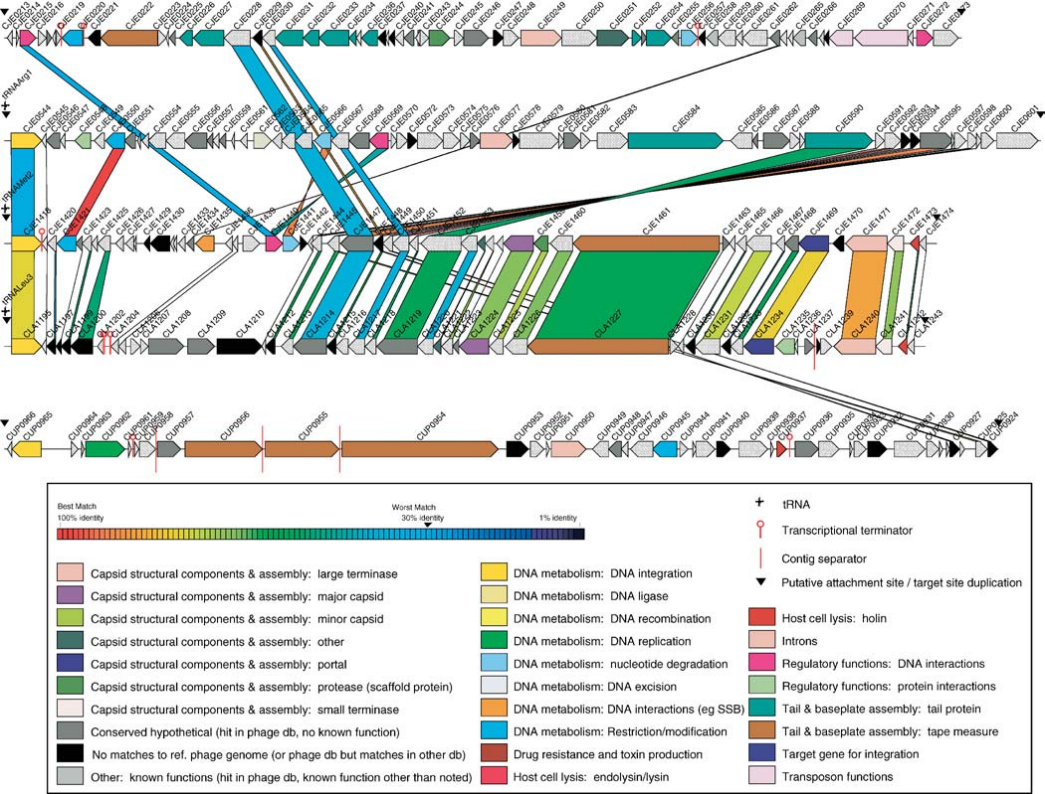
Linear Representations of Prophage Regions Regions are (from top to bottom): CMLP1, CJIE2, CJIE4, CLIE1, and CUIE1. Colors of ORFs are indicated in the key by putative phage function. Connecting lines represent those ORFs whose protein sequences match at a BLASTP of 30% identity or better. These lines do not indicate the coordinates of match, merely that there is a match.

The major difference between the C. jejuni NCTC 11168 and C. jejuni RM1221 genomes is the presence within the strain RM1221 genome of four large integrated elements (Figures 3 and S3). This characteristic has been observed in whole-genome intra-species comparisons of both Gram-positive and Gram-negative microorganisms [38,39,40,41,42]. The first element, *Campylobacter* Mu-like phage (CMLP1) (30.5% G+C content), located upstream of *argC* (CJE0275), encodes several proteins with similarity to bacteriophage Mu and other Mu-like prophage proteins [43], including putative MuA and MuB transposase homologs. Another feature consistent with the identification of CMLP1 as a novel Mu-like prophage is the presence of terminal 5′-TG-3′ dinucleotides flanked by a five-base direct repeat ( TATGC). Preliminary results suggest that this prophage is inducible with mitomycin C and that other C. jejuni strains harbor a related prophage (unpublished data). Genetic manipulation of this phage could yield useful molecular tools analogous to the Mu derivatives for the construction of random gene fusions or mini-Mu elements for in vivo cloning. Although this Mu-like prophage contains no characterized virulence determinants, it could potentially alter pathogenicity or other phenotypes via insertional inactivation.

In contrast to CMLP1, C. jejuni RM1221 integrated elements 2 and 4 (CJIE2 and CJIE4) have integrated into the 3′ end of arginyl- and methionyl-tRNA genes, respectively. Several ORFs predicted to encode phage-related endonucleases, methylases, or repressors are present within these elements; however, unlike CMLP1, few ORFs encoding phage structural proteins were identified within CJIE4. CJIE4 is similar to a putative prophage contained within the C. lari RM2100 genome (C. lari integrated element 1 [CLIE1]); 66% (35/53) of predicted proteins have BLASTP matches (*p*-value ≤ 10^−5^; identity ≥ 30%) (Figure 3). CLIE1 is integrated into a leucinyl-tRNA. The inability to identify matches to major capsid, portal, and scaffold protease proteins within CJIE2 or C. upsaliensis RM3195 integrated element 1 (CUIE1) suggests that they represent either intact prophages with novel head morphogenesis proteins, satellite phages, or nonfunctional prophages or genomic islands.

The absence of any phage-related ORFs within CJIE3 (located within an arginyl-tRNA), suggests that CJIE3 is not a prophage but rather a genomic island or integrated plasmid. Seventy-three percent (45/62) of the CJIE3 predicted proteins are similar to predicted proteins encoded on the C. coli RM2228 megaplasmid (pCC178) (Figure S4; see below), suggesting that CJIE3 was plasmid-derived. However, the observed lack of synteny between CJIE3 and the C. coli RM2228 megaplasmid suggests that CJIE3 was not derived from pCC178 but possibly from a related *Campylobacter* megaplasmid. Although most of the ORFs contained within CJIE3 encode hypothetical proteins (23% 14/62), many are similar to proteins encoded within the 71-kb H. hepaticus ATCC 51449 genomic island (HHGI1), suggesting this genomic island could also be plasmid-derived [44]. Furthermore, 33% (23/70) of HHGI1 proteins match pCC178-encoded proteins.

Bacteriophages are vehicles for the lateral or horizontal movement of genes that can increase bacterial fitness [45,46]. Additionally, it has been demonstrated that bacteriophage-carried genes can play a role in many aspects of bacterial virulence (adhesion, invasion, host evasion, and toxin production) [47]. Though only one of the *Campylobacter* prophages (CMLP1) has been shown to be inducible, we cannot predict whether the other putative prophages or plasmid-like element can be excised. Because the majority of ORFs that lie within prophage regions are hypothetical proteins, we are unable to deduce any putative functions from them; however, we cannot rule out possible functions that either directly impact virulence or increase the fitness of the host in a particular environment.

### Plasmids

C. coli RM2228 and C. lari RM2100 each contain a single plasmid (pCC178; approximately 178 kb, and pCL46, approximately 46 kb, respectively), whereas C. upsaliensis RM3195 contains two plasmids (pCU3, approximately 3.1 kb, and pCU110, approximately 110 kb; Tables 1 and S1). In the current study, neither C. jejuni isolate harbors a plasmid; however, a C. jejuni virulence plasmid, pVir from C. jejuni strain 81–176, was previously sequenced and shown to play a role in pathogenesis [48]. The coding regions of pVir are entirely in one orientation except for a single coding region, which is uncharacteristic for a plasmid of this size. The coding regions of pCU110 and pCL46, like pVir, show a similar coding strand bias. In pCC178, the lack of coding region bias may be explained by the presence of antibiotic resistance genes (Tables 2 and S2) flanked by putative mobile genetic elements. Only the 3.1-kb plasmid of C. upsaliensis RM3195 (pCU3) has a defined plasmid replication region. The single-stranded binding (Ssb) proteins are conserved among all of the plasmids, alluding to a common evolutionary origin; however, the nickase proteins on the plasmids are not conserved, suggesting that nickase may be specific to the plasmid or strain.

**Table 2:**
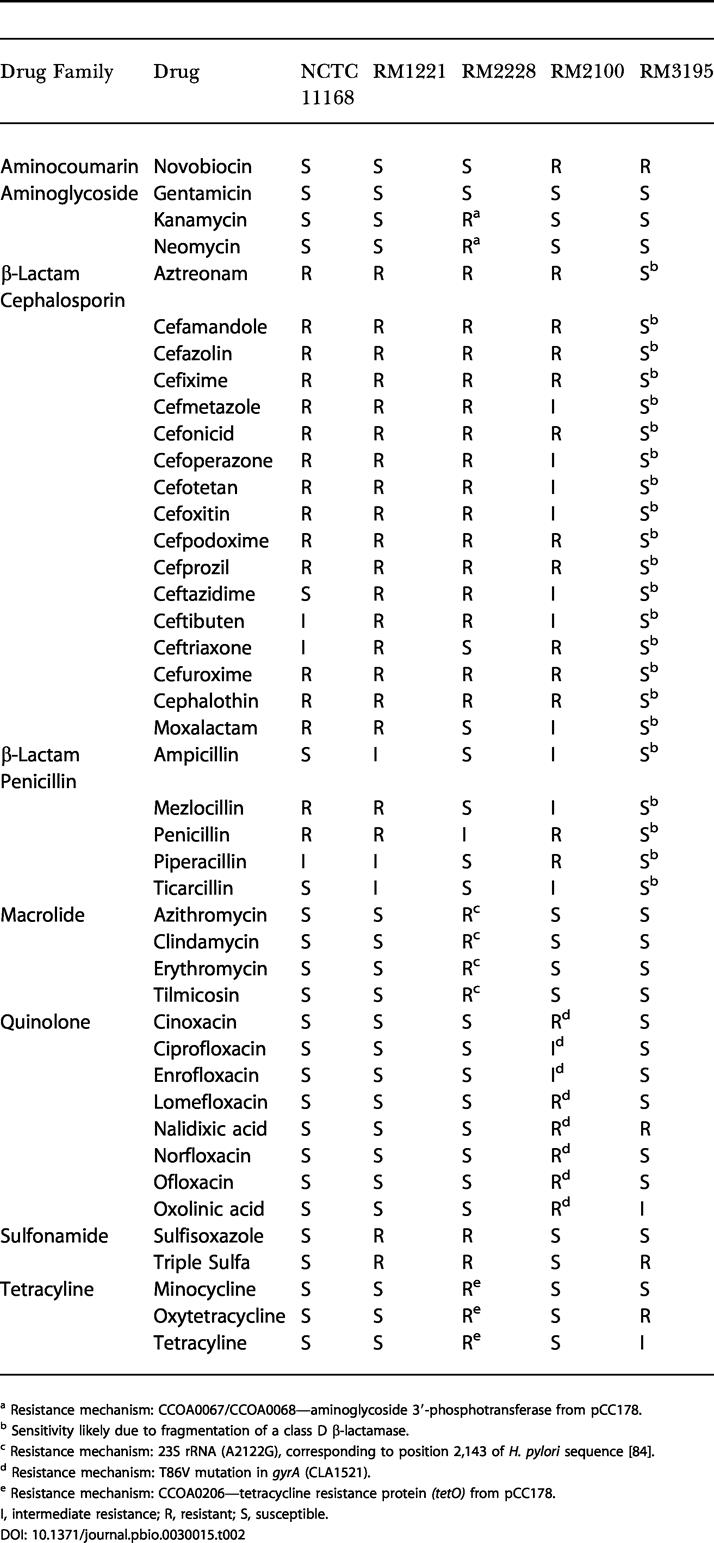
Relevant Drug Resistance Profiles

^a^ Resistance mechanism: CCOA0067/CCOA0068—aminoglycoside 3′-phosphotransferase from pCC178

^b^ Sensitivity likely due to fragmentation of a class D β-lactamase

^c^ Resistance mechanism: 23S rRNA (A2122G), corresponding to position 2,143 of H. pylori sequence [84]

^d^ Resistance mechanism: T86V mutation in *gyrA* (CLA1521)

^e^ Resistance mechanism: CCOA0206—tetracycline resistance protein *(tetO)* from pCC178

I, intermediate resistance; R, resistant; S, susceptible

One conserved feature of all of the large *Campylobacter* plasmids is the presence of a Type IV secretion system (T4SS), possibly involved in conjugative plasmid transfer or secretion of virulence factors [49] (Figure S4). The plasmid-encoded T4SSs in the non–C. jejuni species are most similar to each other based on synteny and amino acid identity; however, they share only synteny with the T4SS encoded by pVir or the Agrobacterium tumefaciens Ti plasmid [50]. The non–C. jejuni plasmid T4SSs may be involved in conjugation rather than secretion of virulence factors because they are more similar to T4SSs known to mobilize DNA than to T4SSs that secrete effectors [50] (Figure S4). Unlike pVir, the other *Campylobacter* plasmids encode proteins similar to VirB2 of the Ti plasmid, which is responsible for pilus formation [49] (Figure S4) and has recently been shown to be essential for DNA transfer, further hinting at a role in DNA mobility [51]. Additionally, pCU110 appears to contain a number of other proteins that are similar to conjugal transfer proteins of other plasmids, which may function independently or in concert with the T4SS to transfer plasmid DNA to donor cells.

### Transposable Elements

Both C. jejuni NCTC 11168 and C. jejuni RM1221 are notable for the apparent absence of intact insertion sequence (IS) elements. With the exception of one copy of a degenerate transposase resembling IS*605,* located between the *tonB* gene and a 5S rRNA gene, their genomes are devoid of IS elements. In contrast, C. coli RM2228 contains five copies of an IS element (IS*Cco1* of the IS*605* family) at three positions in the chromosome and at least two positions in the megaplasmid pCC178, hinting at recent acquisition and transposition competence. Both the C. upsaliensis RM3195 and C. lari RM2100 pseudomolecules lack the *tonB*–5S rRNA locus; however, since these are not closed genomes, we cannot accurately assess the status of the IS*605* family in these genomes.

### CRISPR Analysis

The chromosomes of all five *Campylobacter* strains in this study were examined for the presence or absence of clustered regularly interspaced short palindromic repeats (CRISPRs) in intergenic regions. A strain was considered CRISPR-positive if it contained two or more direct repeats of a 21-bp or larger DNA segment separated by unique spacer sequences of a similar size. We identified CRISPR elements in only C. jejuni NCTC 11168 and C. jejuni RM1221. However, a previous study found that CRISPR elements are sometimes detectable in C. coli [52]. Also consistent with the previous study, the two strains of C. jejuni examined here can be differentiated by both the unique sequence of the spacer sequences (Figure S5) and the number of CRISPR repeats in the element (five in C. jejuni NCTC 11168 and four in C. jejuni RM1221). It is noteworthy that the previous study did not include C. lari or *C. upsaliensis,* which appear not to contain CRISPR elements, unless they are in a different region of the genome from the C. jejuni CRISPRs and are in unsequenced areas. This further demonstrates the limited utility of CRISPRs in genotyping studies of *Campylobacter* species.

### Restriction–Modification Systems

The Type I restriction–modification (RM) loci from 65 C. jejuni strains have been characterized previously [53]. In contrast to the *C. jejuni, C. coli,* and C. lari strains sequenced in this study, the C. upsaliensis RM3195 genome is predicted to contain at least three Type I RM loci (Table S3). C. upsaliensis RM3195 also contains a putative fourth locus where the *hsdR* gene is absent. The sequenced genomes of the C. jejuni strains NCTC 11168 and RM1221, C. coli RM2228, and C. lari RM2100 encode few Type II or Type III RM systems. C. upsaliensis RM3195 encodes one putative Type II and two putative Type III restriction enzymes. In addition, C. upsaliensis RM3195 encodes 15 putative adenine- or cytosine-specific DNA methyltransferases. It is noteworthy that the sequenced genome of H. hepaticus ATCC 51449, like C. jejuni RM1221, C. coli RM2228, and C. lari RM2100, has a paucity of RM loci [44] and would therefore be considered “*Campylobacter*-like” whereas C. upsaliensis RM3195 would be considered “*Helicobacter* *Pylori*-like” with respect to RM systems. At least four of the C. upsaliensis RM3195 RM systems lie within regions of atypical nucleotide composition, suggesting recent horizontal transfer as selfish mobile elements [54].

Diversity within the *Campylobacter* RM systems has implications for *Campylobacter* biology, specifically DNA uptake and phage infection. *Campylobacter* spp. are naturally competent [55], and horizontal gene transfer via natural transformation is thought to play an important role in the evolution of C. jejuni [56]. Natural competence, as well as experimental introduction of DNA by electroporation, would be influenced presumably by host RM systems. Indeed, strain-specific differences in competence have been noted in *Campylobacter* [1,57]. RM system variation would also impact infection by both lytic and lysogenic bacteriophages. Future studies will be able to determine the functional status of the RM systems and their role in natural competence and phage restriction.

### *Campylobacter* Metabolism

There have been relatively few studies of the metabolic capabilities of *Campylobacter* spp., but they are known to have a respiratory type of metabolism, with some strains growing under both aerobic and anaerobic conditions [58,59]. Carbohydrates in general are not utilized. Comparative analysis of the genomes of C. jejuni RM1221, C. coli RM2228, C. lari RM2100, and C. upsaliensis RM3195 revealed that these species have very similar metabolic profiles, with the main variation being the presence of a complete or partial tricarboxylic acid cycle (Figure S6). In C. jejuni RM1221, the tricarboxylic acid cycle appears to be intact and most likely serves a dual role of generating biosynthetic compounds and providing intermediates that feed into electron transport. C. coli RM2228, C. upsaliensis RM3195, and C. lari RM2100 apparently lack a succinate dehydrogenase, and none of the strains appear to encode SucAB (oxoglutarate dehydrogenase). All four sequenced strains have pathways for the metabolism and biosynthesis of a number of amino acids (Figure S6), and acetate, formate, and lactate appear to be the main end products of carbon metabolism. Preliminary Biolog data demonstrate differences in substrate utilization patterns across the *Campylobacter* strains in this study. C. jejuni RM1221, C. coli RM2228, and C. lari RM2100 all respire in the presence of arabinose, fucose, and formic and lactic acid. In addition, C. jejuni RM1221 respires in the presence of fructose, mannose, hydroxybutyric acid, asparagine, and aspartic acid, in contrast to the other species. These observed phenotypic differences from the preliminary Biolog data may be a reflection either of the conditions under which the substrates were tested or of C. jejuni having pathways that are lost in the other strains. Because of the lack of complete genomes from the other strains, we cannot say with confidence what the reason is for the observed differences, but variable patterns in substrate utilization by *Campylobacter* species have previously been described [60]. Some of these substrate utilization differences might stem from strain- and species-specific ORFs present in these isolates, or from simple gene mutations that cannot be detected at the genome level. In C. jejuni NCTC 11168, for example, the inability to grow on sugars that are added to the growth medium is felt to be a reflection of the missing phosphofructokinase that is necessary for glycolysis [28]. Interestingly, for all the ɛ-Proteobacteria included in this study, no phosphofructokinase could be identified except for *W. succinogenes,* enabling *Wolinella* to metabolize a wider range of carbohydrates than *Campylobacter*.

### Chromosomally Encoded Protein Secretion Systems

The five *Campylobacter* strains analyzed in this study have the Sec-dependent and Sec-independent (twin-arginine translocation “TAT”) protein export pathways for the secretion of proteins across the inner/periplasmic membrane. In addition, *Campylobacter* has the signal recognition particle pathway. We have found no evidence for chromosomally encoded *lol,* Type III, or Type IV secretion systems other than the flagellar export apparatus [61]. In all five strains, there are putative proteins that comprise components of a transformation system with similarity to Type II secretion systems [62]. A putative pre-pilin peptidase and several putative pseudopilins have been identified based on BLASTP similarity or the presence of an N-terminal pre-pilin peptidase cleavage signal (Table S4). The two-partner secretion/single accessory pathway [63] is used by Gram-negative bacteria to secrete adhesins and cytolysins [63]. There are undisrupted copies of putative pore-forming single accessory factors (generically termed TpsB homologs) in C. coli RM2228 (CCO0190), C. lari RM2100 (CLA0150), and C. jejuni NCTC 11168 (Cj0975); however, CCO1305 in C. coli and CJE0841–CJE0843 and CJE1056 in C. jejuni RM1221 are disrupted (Figure S7). It is unclear whether these disruptions are real in the unfinished genomes or whether there would be any consequence for the disruption in C. jejuni RM1221.

### Virulence

The pathogenic mechanisms responsible for acute intestinal infections by *Campylobacter,* although still poorly understood, are thought to involve adherence, cellular invasion, and toxin production, but not all clinical isolates of C. jejuni are able to invade cultured human cells or produce defined toxins [64]. However, a common feature of *Campylobacter* infectious enterocolitis is a localized acute inflammatory response that can lead to tissue damage and may be responsible for many of the clinical symptoms [64].

Motility is the major factor that has been implicated directly in intestinal colonization [65]. Of the 580 ORFs conserved between the *Campylobacter* and *Helicobacter* species included in this study (see Figure 1C), 27 ORFs involved in flagellar biosynthesis and function were conserved between *Campylobacter* and *Helicobacter*. Another set of 18 ORFs involved in chemotaxis and motility was found to be conserved across the *Campylobacter* strains, but with no bidirectional match in *Helicobacter* (criteria: *p*-value ≤ 10^−5^, identity ≥ 35%, match lengths of at least 75% of the length of both query and subject sequence), emphasizing the importance of bacterial motility and adhesion for virulence [66].

Two-component regulatory (TCR) systems are used commonly by bacteria to respond to specific environmental signals. We identified five TCR systems (pairs of adjacent histidine kinase and response regulator genes) that appear to be conserved across the *Campylobacter* spp.: CJE0968–CJE0969, CJE1357–CJE1358, CJE1361–CJE1362, *racR–racS* (CJE1397– CJE1398), and CJE1664–CJE1665. In addition, another four putative response regulator genes (CJE0746, CJE0404, CJE1168, and CJE1780) and one putative histidine kinase gene (CJE0884) could be found in the finished C. jejuni genomes. Brás et al. [67] showed that the RacR–RacS system is involved in a temperature-dependent signaling pathway and is required for the organism to colonize the chicken intestinal tract. The high degree of conservation of these ORFs suggests an importance in the *Campylobacter* pathogenicity, not surprising given the likely exposure of the bacteria to temperature stress during the infectious process.

Adherence of C. jejuni to epithelial cells is mediated by multiple adhesins, including CadF (CJE1651), PEB1 (CJE0997–CJE1000), JlpA (CJE1065), and a 43-kDa major outer membrane protein (CJE1395). Fibronectin (FN) has been implicated in C. jejuni adherence to epithelial cells via the protein CadF [68]. In addition to CadF, we found two putative FN-binding proteins (CJE1415 and CJE1538) that are conserved across the five *Campylobacter* strains. The FN host cell-surface receptor is the α5β1 integrin. In intact epithelia, α5β1 integrins are restricted to the basolateral membrane and thus are not available for interaction with luminally positioned microbial pathogens [69]. However, Monteville et al. showed that adherence and internalization of C. jejuni were greatly increased by exposure of cellular basolateral surfaces, and that FN was the receptor [70]. This suggests that C. jejuni invasion preferentially occurs via a paracellular route, rather than via an intracellular route. Additionally, inspection of loci adjacent to putative TpsB proteins revealed two intact filamentous hemagglutinin (FHA)–like adhesions: in C. lari RM2100, CLA0151, and in C. coli RM2228, CCO1312. The regions upstream of the remaining TpsB-like proteins have fragmented adhesion-like ORFs (Table 1; Figure S7). Only C. lari RM2100 has both an undisrupted TpsB-like transporter (CLA0150) and an adjacent putative FHA-like adhesion (CLA0151), which, if functional, could enable C. lari RM2100 to attach to cell surfaces.

Cytolethal distending toxins from enteropathogenic Escherichia coli have been shown to disrupt the barrier function of host intestinal epithelial tight junctions [71]. The three cytolethal distending toxins A, B, and C (CJE0075, CJE0074, and CJE0073) were conserved across the five *Campylobacter* strains. In addition, C. lari RM2100 encodes a single peptide (CLAA0034) in pCL46 that is similar to the *Yersinia* invasin proteins that enable *Yersinia* to penetrate host cells [72], suggesting that this C. lari strain might also have the ability to penetrate host cells.

### Identification of a Novel *Campylobacter* Putative Virulence Locus

Examination of the C. upsaliensis RM3195 sequence revealed a putative *licABCD* (CUP0277–CUP0274) locus with varying, but significant, identity to genes present in Haemophilus influenzae [73], commensal *Neisseria* species [74], and Streptococcus pneumoniae [75]. *licABCD* genes in these microorganisms encode proteins involved in the acquisition of choline (*licB,* CUP0276), synthesis of phosphorylcholine (PCho) (*licA,* CUP0277; *licC,* CUP0275), and transfer of PCho (*licD,* CUP0274) to LOS or teichoic/lipoteichoic acids to facilitate attachment to host cells [74]. Preliminary studies indicate that other strains of C. upsaliensis from South Africa also contain *licA* (unpublished data). It is noteworthy that *licA* expression in *Haem. influenzae* is regulated by variation in the number of intragenic tandem tetranucleotide repeats ( CAAT) at the 5′ end, resulting in translational on/off synthesis of PCho and expression on LOS [76]. A poly G tract within the *licA* gene (bp 132–146) of C. upsaliensis RM3195 probably regulates synthesis of PCho and decoration of LOS by a similar mechanism.

### Hypervariable Homopolymeric Tracks

The presence of the homopolymeric repeat sequences in the genome of C. jejuni NCTC 11168 has been described [28]. However, in comparing these five *Campylobacter* strains, a number of other phenomena related to these repetitive regions were observed. First, when a homopolymeric repeat region was associated with a potential coding region, the base mostly included in the repeated region on the coding strand was G, resulting in poly-glycine, not poly-proline, in the peptide. Secondly, the C. upsaliensis RM3195 genome contains nearly three times as many variable homopolymeric repeats (22) as C. jejuni RM1221 (8), seven times as many as C. lari RM2100 (3), and 22 times as many as C. coli RM2228 (1) (Table 1). These varied C. upsaliensis RM3195 poly G:C tracts come from a pool of almost five times as many total poly G:C tracts (Table 1) as C. jejuni RM1221 and C. coli RM2228, and nearly ten times as many total poly G:C tracts as C. lari RM2100. Of these 22 varied poly G:C tracts, 11 (50%) are strain-specific (Tables S5 and S6). It appears that excess variable poly G:C tracts are due to the presence of unique ORFs; however, it is unclear as to why C. upsaliensis RM3195 contains so many more total homopolymeric repeated regions, since only 61 of the 209 regions are within unique ORFs. These variable regions encode a combination of hypothetical, cell envelope, and virulence-associated ORFs (Table S6), which in other pathogenic bacteria has been shown to be the molecular basis of lipopolysaccharide phase variation [77], has been used to identify novel virulence genes in *Haem. influenzae* [78], and has been speculated to have a similar role in C. jejuni [28]. However, these observed differences could be the result of different culturing conditions prior to library construction.

### LOS and EP Biosynthesis

LOSs and EPs are important surface structures in C. jejuni that function in the interactions of the organism with the environment. Interesting aspects of C. jejuni LOSs are their molecular mimicry of host gangliosides and their presumed roles in evasion of host immune responses and autoimmunity [79], decreased immunogenicity [80], and attachment and invasion [48]. The capsule of C. jejuni 81–176 has been reported to have a role in increasing serum resistance, invasion of cell lines, and surface hydrophilicity [81].

The LOS biosynthesis loci of all sequenced *Campylobacter* spp. are organized as previously observed in other C. jejuni strains [82]. At either end of the loci are the heptosyltransferase genes, *waaC* and *waaF,* that surround regions exhibiting significant variation in ORF content. Thus, these organisms likely synthesize novel LOS structures [82]. In particular, the LOS of C. jejuni RM1221 is distinct from the LOS of NCTC 11168, as seen on polyacrylamide gels, in that it possesses three LOS bands while NCTC 11168 possesses only one (unpublished data). Two LOS genes from C. jejuni RM1221 possess homopolymeric G:C tracts that may explain the additional bands. Comparison of the LOS genes from the sequenced *Campylobacter* spp. with those from C. jejuni strains that produce ganglioside mimics [29] demonstrates that these four strains do not possess the genes involved in the synthesis of *N*-acetylneuramic (sialic) acid or the associated sialic acid transferase, and are not likely to produce ganglioside mimics. Within the LOS loci of C. lari RM2100 and C. upsaliensis RM3195, there are ORF clusters that have homologs in NCTC 11168 that are unrelated to LOS biosynthesis. It is unclear what role this genomic reorganization plays in the biosynthesis of LOS.

C. jejuni RM1221, C. coli RM2228, and C. lari RM2100 possess *kps* orthologs like the EP locus of C. jejuni NCTC 11168 that are involved in polysaccharide export; however, many putative EP biosynthesis genes from C. jejuni RM1221 and C. coli RM2228 are unique to these strains. The *kps* orthologs are present in C. upsaliensis RM3195, but they are not clustered with other polysaccharide biosynthetic genes as observed in the other strains. Specifically, there are three clusters of EP genes: CUP0615–CUP0619, CUP1248–CUP1270, and CUP1328–CUP1329. The second cluster contains many ORFs that are unique to C. upsaliensis (Table S5), including two of the three copies of a putative GDP-fucose synthetase (CUP1255, CUP1257, and CUP1258). Only C. jejuni strains (Cj1428c and CJE1612) and C. upsaliensis RM3195 encode this enzyme. Of these GDP-fucose synthetases, only CUP1257 was shown to contain variable poly G tracts (Table S6).

### Antibiotic Resistance

The sequenced *Campylobacter* strains have adapted or acquired many mechanisms of antibiotic resistance (Tables 2 and S2). All strains are resistant to cloxacillin, nafcillin, oxacillin, sulfamethoxazole/Tm, trimethoprim, and vancomycin, and this resistance is likely inherent to all *Campylobacter* spp. (Table S2). Every strain but C. upsaliensis RM3195 is resistant to most β-lactam antibiotics. This general lack of resistance to β-lactam antibiotics for RM3195 is likely due to the disruption of a class D β-lactamase matching GenBank accession AAT01092 (CUP0345), which was found as an intact single copy in NCTC 11168 (Cj0299), RM1221 (CJE0344), and RM2100 (CLA0304). The corresponding sequence in C. coli RM2228 may reside in unsequenced regions. Only C. lari RM2100 was resistant to a broad range of quinolone/fluoroquinolone antibiotics (Table 2). This broad quinolone/fluoroquinolone resistance is most likely the result of adaptation via a mutation of DNA gyrase *(gyrA)* that changed codon 86 from threonine to valine [83]. The macrolide antibiotics azithromycin, clindamycin, erythromycin, and tilmicosin were effective against all but C. coli RM2228. This is likely due to a mutation in all three copies of the 23S rRNA (A2122G), corresponding to position 2,143 of the H. pylori sequence [84]. C. coli RM2228 has acquired resistance to the aminoglycosides kanamycin and neomycin, tetracycline, oxytetracycline, minocycline, and presumably hygromycin B (but not gentamicin) from the megaplasmid pCC178 (Table 2). It is possible that C. coli has acquired resistance to macrolides and tetracyclines as a result of the application of these drugs during poultry production. The resistance of C. upsaliensis RM3195 to oxytetracycline and its intermediate resistance to tetracycline may be due to the action of multi-drug efflux pumps or a novel mechanism, since there is no evidence for tetracycline resistance genes [85], and there are no known mutations in the 16S rRNA [86]. Similarly, no known mutations in *gyrA* or *gyrB* were found in C. upsaliensis RM3195 to explain the resistance to nalidixic acid [83] and novobiocin [87]. There were no obvious known mutations of dihydropteroate synthase *(folP)* [88] to explain the observed variable resistance to sulfonamide-class drugs (Table 2). Rifampin resistance was observed in all strains but C. lari RM2100, but was not due to the classic mutations in the β subunit of RNA polymerase [89].

### Conclusions

The comparison of five sequenced *Campylobacter* genomes has provided the core genetic blueprint of the genus. Although the blueprint reveals obvious differences in genome structure and content, additional epidemiological data are needed to correlate these differences, and other, more elusive differences (e.g. differences in regulation and point mutations), with differences in virulence. Some obvious differences were the presence of drug resistance genes that may have been the result of adaptation in the animal production environment, where antibiotics are frequently used to eliminate bacterial infections. It is anticipated that the analysis of the *Campylobacter* genomes presented here will lay the foundation for the development of systems for fingerprinting strains for phylogenetics, epidemiology, and source tracking, as well as the development of alternative treatments for controlling *Campylobacter* in food production and in human infection.

## Materials and Methods

### 

#### Strain isolation and propagation

C. jejuni strain RM1221 ( ATCC BAA-1062) was isolated from the skin of a retail chicken using methods modified from those described previously for isolation of *Campylobacter* from chicken products [31]. C. coli strain RM2228 ( ATCC BAA-1061) was isolated from a chicken carcass obtained from an inspected slaughter plant. A rinse sample was streaked on 5% sheep blood agar plates, and the plates were incubated at 37 °C for 48 h under an atmosphere of 5% O_2_, 10% CO_2_, and balance N_2_. An isolated single colony was picked and maintained on sheep blood agar plates. Three rounds of mixing and sonication of single colony picks were done as described [31]. C. lari strain RM2100 ( ATCC BAA-1060) is a human isolate obtained from the Centers for Disease Control and Prevention, Atlanta, Georgia, United States (CDC strain D67, “case 6” [32]). The strain was maintained on *Brucella* agar amended with 5% (v/v) laked horse blood (Hema Resource and Supply, Aurora, Oregon, United States). Three rounds of mixing and sonication of single colony picks were done as described [31]. C. upsaliensis strain RM3195 ( ATCC BAA-1059) was obtained from the feces of a 4-y-old boy confirmed clinically to have Guillain-Barré syndrome. The isolation procedure involved a filtration method with selection of *Campylobacter* cells in diluted feces by their migration through a 0.6-μm membrane filter and subsequent growth on nonselective medium [33].

#### Genome sequencing

The four species of *Campylobacter* were sequenced by the random shotgun method [38]. The genome of C. jejuni RM1221 was sequenced to closure, whereas the genomes of strains C. lari RM2100, C. coli RM2228, and C. upsaliensis RM3195 were sequenced to 8-fold coverage of an estimated 1.8-Mbp genome. Briefly, one small insert plasmid library (1.5–2.5 kb) and one medium insert plasmid library (10–12 kb) were constructed for each strain (except RM1221, which had only a small insert library) by random nebulization and cloning of genomic DNA. In the random sequencing phase, 8-fold sequence coverage was achieved from the two libraries (sequenced to 5-fold and 3-fold coverage, respectively). The sequences from the respective strains were assembled separately using TIGR Assembler [90] or Celera Assembler [91]. All sequence and physical gaps for C. jejuni RM1221 were closed by editing the ends of sequence traces, primer walking or transposon-primed sequencing [92] on plasmid clones, and combinatorial PCR followed by sequencing of the PCR product. The correct nucleotide sequences for repetitive regions greater than the maximum insert size of 2.5 kb (i.e., rRNA operons) for C. jejuni RM1221 were confirmed by sequencing PCR products that spanned each repeat unit. Pseudomolecules for the draft sequences were constructed using NUCmer [93] and BAMBUS [38,94] as previously described [38].

#### Ambiguity rate

The ambiguity rate for the unfinished genomes was determined using the following procedure. First, the consensus of the contigs was recalled using the consensus caller included in the AutoEditor package (http://www.tigr.org/software/autoeditor/) [95] by executing “autoEditor—noedit” on the final contigs. This step was necessary because the contigs as produced by the Celera Assembler were made with a consensus caller which does not assign ambiguity codes, but instead assigns a base call arbitrarily in the event of a tie or near tie situation. The AutoEditor consensus caller recomputes the consensus at each position and assigns an ambiguity code if there is sufficient conflicting information. Using a custom script, a count was made of both the overall number of positions and the number of ambiguous positions with at least the specified depth of coverage. This was necessary because the depth of coverage in the assemblies is not uniform, but directly influences the ambiguity rate. For example, under the AutoEditor ambiguity model, there are no ambiguous positions at 1-fold coverage. The ambiguity rate is then reported as the ratio of the two counts, as a close approximation to the error rate of the true consensus sequence.

#### Annotation

An initial set of ORFs that likely encode proteins was identified using GLIMMER [96], and those shorter than 90 bp or those with overlaps were eliminated. ORFs were searched against a nonredundant protein database; frameshifts and point mutations were processed only for C. jejuni RM1221 [38]. Two sets of hidden Markov models were used to determine ORF membership in families and superfamilies [38].

#### Comparative genomics

For the identification of species-specific (Table S7) and strain-specific (Table S5) ORFs, all predicted proteins (excluding pseudogenes) from the four TIGR-sequenced *Campylobacter* genomes and C. jejuni NCTC 11168 [28] were searched against an in-house database composed of 734,467 protein sequences encoded by 19 archaeal, 192 bacterial, 146 eukaryotic, three phage, and 17 virus chromosomes, as well as 145 plasmid, 29 mitochondrial, 17 plastid, and three nucleomorph genomes, using WU-BLASTP (http://blast.wustl.edu) [97]. To identify genus-specific ORFs, the protein sequences from the above five *Campylobacter* genomes plus three *Helicobacter* genomes (H. pylori 26695 [98], H. pylori J99 [99], and H. hepaticus ATCC 51449 [44]) and the genome of W. succinogenes DSMZ1740 [100] were compared. Specifically, only bidirectional best matches that met the following prerequisites were scored: a *p*-value less than or equal to 10^−5^, identity of 35% or more, and match lengths of at least 75% of the length of both query and subject sequence. Match tables were created that were later used to generate the Venn diagrams (Tables S8 and S9). Novel ORFs encoded proteins that had no WU-BLASTP match. Regions of synteny were identified by first finding the maximum unique matches with a minimum length of five amino acids using PROmer, followed by visualization of the data using MUMmerplot (http://www.tigr.org) and Gnuplot version 4.0 (http://www.gnuplot.info/).

#### MLST and FlaA SVR typing

The MLST of C. jejuni RM1221 was determined by searching the nucleotide sequences of aspartate ammonia-lyase (*aspA,* CJE0082), glutamine synthetase type I (*glnA,* CJE0798), citrate synthase (*gltA,* CJE1851), serine hydroxymethyltransferase (*glyA,* CJE0451), phosphoglucosamine mutase (*pgm/glmM,* CJE0409), transketolase (*tkt,* CJE1817), and ATP synthase F1 alpha subunit (*uncA/atpA,* CJE0100) on the PubMLST Web site (http://pubmlst.org/) [101]. The sequence of the C. jejuni RM1221 FlaA SVR was found by searching the *flaA* (CJE1528) nucleotide sequence using the sequence of primers FLA242FU and FLA625RU [34]. This nucleotide sequence was used to query the *flaA* allele database (http://phoenix.medawar.ox.ac.uk/flaA/) to elucidate the FlaA SVR type [34,102].

#### Phylogenetic analysis

The programs SEQBOOT, DNAML, PROML, and CONSENSE are part of the PHYLIP version 3.62 package (http://evolution.genetics.washington.edu/phylip.html, http://fink.sourceforge.net/) [103]. Both the 16S rRNA and concatenated protein trees were rooted to the δ-Proteobacterium Desulfovibrio vulgaris subsp. *vulgaris* strain Hildenborough sequences [104]. One hundred bootstrapped datasets were generated using the SEQBOOT program, and consensus trees were determined using CONSENSE. The final trees with preserved branch lengths were computed with the user tree option of DNAML and PROML.

16S rRNA trees were generated by first creating a multiple alignment using the “PHYLIP Interface” option of the Ribosomal Database Project release 8.1 (http://35.8.164.52/cgis/phylip.cgi, which aligns user-supplied 16S rRNA sequences against the Ribosomal Database Project alignment. The produced alignment was trimmed and gaps removed using an in-house PERL (http://www.perl.org) script. Maximum-likelihood trees were generated using DNAML (R = gamma-distributed rate of variation [coefficient of variation, 1.41; four hidden Markov model rate categories] and S = NO).

Protein trees were generated from concatenated multiple alignments of 12 conserved proteins (initiation factor 2 [InfB]; elongation factors G [FusA] and Tu [Tuf]; ribosomal proteins L2 [RplB], S5 [RpsE], S8 [RpsH], and S11 [RpsK]; DNA topoisomerase I [TopA]; signal recognition particle protein [Ffh] [36]; DNA gyrase B subunit [GyrB]; GTP-binding protein LepA; and CTP synthase [PyrG] [37]). Each protein was aligned separately using CLUSTALW version 1.82 [105], using the slow, more accurate option. The alignments were trimmed to remove gaps using BELVU version 2.16 (http://www.cgb.ki.se/cgb/groups/sonnhammer/Belvu.html). Each organism's aligned sequences were concatenated using an in-house PERL script. Maximum-likelihood trees were generated using PROML (P = Jones-Taylor-Thornton model of change between amino acids, R = gamma-distributed rate of variation [coefficient of variation, 1.41; four hidden Markov model rate categories], and S = NO).

#### Hypervariable homopolymeric G or C tracts

Hypervariable homopolymeric G or C tracts were identified by analyzing the underlying sequences for each nucleotide within a tract of six or more G or C nucleotides. A hypervariable tract was considered of high quality if its underlying sequence comprised at least three sequencing reads with an average Phred score greater than 30 [106].

## Supporting Information

Figure S1 Circular Representation of the Closed C. jejuni RM1221 Genome Each concentric circle represents genomic data and is numbered from the outermost to the innermost circle. Refer to the key for details on color representations. The first and second circles represent predicted ORFs on the plus and minus strands, respectively. The third circle shows the GC-skew. The fourth circle depicts genetic loci with characteristics or functions of interest: CRISPRs, DNA competence, EP, LOS, prophage and genomic island regions, motility, repeats, and Type I restriction/modification regions. The fifth circle demarcates *C. jejuni–*specific and C. jejuni RM1221–specific ORFs. The sixth circle plots atypical regions (χ^2^ value). The seventh circle denotes tRNA, rRNA, and sRNA (tmRNA and 4.5S RNA) loci.
(2.6 MB EPS).

Figure S2 Linear Illustration of C. jejuni Genome Comparisons (274 KB PDF).

Figure S3 Comparison of Plasmid-Like Genomic Islands of C. jejuni RM1221 CJIE3 (top linear figure) and H. hepaticus ATCC 51449 HHGI1 (bottom line) against pCC178 megaplasmid of C. coli RM2228 (middle line). Colors of ORFs are indicated in the key by putative function. Connecting lines represent those ORFs whose protein sequences match at a BLASTP of 30% identity or better. These lines do not indicate the coordinates of match, merely that there is a match.
(76 KB PDF).

Figure S4 T4SS Is Shared among the Large *Campylobacter* Species Plasmids but Is Not the Same as C. jejuni T4SS (A) shows a conceptual diagram indicating where each of the proteins thought to be involved in the T4SS interact. Each corresponding loci is color-coded in each of the plasmids.
(B) The T4SS in each of the plasmids demonstrates that a number of the core proteins are conserved in all of the *Campylobacter* plasmids; however, the non–C. jejuni plasmids contain a structure that is more similar to the Agrobacterium tumefaciens T4SS. (In the *Campylobacter* plasmids, black ORFs are those not directly involved in the T4SS; however, many are similar to plasmid transfer proteins).
(5.3 MB EPS).

Figure S5 DNA Sequences of the CRISPR Elements Found in the Two Strains of *C. jejuni,* RM1221 and NCTC 11168 The characters in italics indicate the 32-bp spacer sequences that are unique to the two strains; the spacer sequences for NCTC 11168 are 1 bp longer than presented by others [52]. The bold characters represent the CRISPR repeat region in RM1221 (*n* = 4) and NCTC 11168 (*n* = 5). The characters in roman typeface indicate regions flanking the repeat region that are identical in the two strains.
(20 KB DOC).

Figure S6 Main Pathways for Metabolism Derived from an Analysis of Five *Campylobacter* Genomes The tricarboxylic (TCA) cycle has major variations based on comparative analysis across the strains (please refer to text). Differences in substrate respiration based on an analysis of Biolog data and species-specific pathways are also presented in the text.
(51 KB PPT).

Figure S7 Putative Two-Partner/Single Accessory Secretion Loci FhaC, the single accessory protein that secretes the Bordetella pertussis FHA across the outer membrane, was used as the query for BLASTP searches against a database containing *Campylobacter* protein sequences. Fragments of single accessory proteins were found as matches in the *Campylobacter* match table (see Table S8). Putative single accessory protein/TpsB family proteins (teal) and putative FHAs/hemolysins (red) are noted, as well as putative proteins with weak matches to metacaspases or toxins (tan). The small red ORFs suggest fragmentation of a larger, full-length ORF.
(1.6 MB EPS).

Table S1 Comparison of *Campylobacter* Species Plasmids (19 KB XLS).

Table S2 Antibiotic Susceptibility Profiles (22 KB XLS).

Table S3 *C. jejuni, C. coli, C. lari,* and C. upsaliensis Restriction-Modification (22 KB XLS).

Table S4 Putative DNA Competence Genes (16 KB XLS).

Table S5 Strain-Specific Genes with Annotations (238 KB XLS).

Table S6 Hypervariable Homopolymeric Sequences Found in *Campylobacter* Genomes (57 KB XLS).

Table S7 C. jejuni–Specific Genes with Annotations (31 KB XLS).

Table S8 Match Table Depicting Bidirectional Best Matches of *Campylobacter* Species (647 KB XLS).

Table S9 Match Table Depicting Bidirectional Best Matches of Sequenced ɛ-Proteobacteria (894 KB XLS).

Table S10 Arg-Gly-Asp, Lipoprotein, Outer Membrane Protein Signal, Secretion Signal, and Transmembrane Motif Results (155 KB XLS).

### Accession Numbers

The nucleotide sequence for the closed genome of C. jejuni RM1221 has been deposited at the DNA Data Bank of Japan (DDBJ; http://www.ddbj.nig.ac.jp/, the European Molecular Biology Laboratory Nucleotide Sequence Database (EMBL; http://www.ebi.ac.uk/embl/, and GenBank (http://www.ncbi.nlm.nih.gov/Genbank/) under accession number CP000025. The whole-genome shotgun projects for the genomes of C. lari RM2100, C. coli RM2228, and C. upsaliensis RM3195 that were sequenced to at least 8-fold coverage were deposited at DDBJ, EMBL, and GenBank under accession numbers AAFK00000000, AAFL00000000 and AAFJ00000000, respectively. The versions described in this paper are the first versions, AAFK01000000, AAFL01000000 and AAFJ01000000, respectively. Additionally, all sequence traces and assemblies were deposited at the National Center for Biotechnology Information assembly archive (http://www.ncbi.nlm.nih.gov/Traces/assembly). The contig separator that was used to create the pseudomolecules for the unfinished genomes is NNNNN TTAATTAATTAANNNNN.

## Abbreviations

CJIE: Campylobacter jejuni RM1221 integrated element
CLIE: Campylobacter lari RM2100 integrated element
CMLP1: *Campylobacter* Mu-like phage
CRISPR: clustered regularly interspaced short palindromic repeat
CUIE: Campylobacter upsaliensis RM3195 integrated element
EP: capsular (extracellular) polysaccharide
FHA: filamentous hemagglutinin
FN: fibronectin
HHGI1: Helicobacter hepaticus ATCC 51449 genomic island
IS: insertion sequence
LOS: lipooligosaccharide
MLST: multilocus sequence type
ORF: open reading frame
PCho: phosphorylcholine
RM: restriction–modification
SVR: short variable region
T4SS: Type IV secretion system
TCR: two-component regulatory

## References

[pbio-0030015-b01] Miller WG, Mandrell RE, Ketley J, Konkel ME (2004). *Campylobacter* in the food and water supply: Prevalence, outbreaks, isolation, and detection. *Campylobacter jejuni*: New perspectives in molecular and cellular biology.

[pbio-0030015-b02] Friedman CR, Neimann J, Wegener HC, Tauxe RV, Nachamkin I, Blaser MJ (2000). Epidemiology of Campylobacter jejuni infections in the United States and other industrialized nations. Campylobacter.

[pbio-0030015-b03] Vandamme P, Nachamkin I, Blaser MJ (2000). Taxonomy of the family *Campylobacteraceae*. Campylobacter.

[pbio-0030015-b04] Lastovica AJ, Skirrow MB, Nachamkin I, Blaser MJ (2000). Clinical significance of *Campylobacter* and related species other than Campylobacter jejuni and C. coli. Campylobacter.

[pbio-0030015-b05] Nachamkin I, Allos BM, Ho T (1998). *Campylobacter* species and Guillain-Barre syndrome. Clin Microbiol Rev.

[pbio-0030015-b06] Willison HJ, O'Hanlon GM (1999). The immunopathogenesis of Miller Fisher syndrome. J Neuroimmunol.

[pbio-0030015-b07] Kist M, Bereswill S (2001). Campylobacter jejuni. Contrib Microbiol.

[pbio-0030015-b08] Glunder G, Petermann S (1989). [The occurrence and characterization of *Campylobacter* spp. in silver gulls (*Larus argentatus*), three-toed gulls *(Rissa tridactyla)* and house sparrows (*Passer domesticus*)]. Zentralbl Veterinarmed B.

[pbio-0030015-b09] Hald B, Pedersen K, Waino M, Jorgensen JC, Madsen M (2004). Longitudinal study of the excretion patterns of thermophilic *Campylobacter* spp. in young pet dogs in Denmark. J Clin Microbiol.

[pbio-0030015-b10] Harvey RB, Young CR, Ziprin RL, Hume ME, Genovese KJ (1999). Prevalence of *Campylobacter* spp isolated from the intestinal tract of pigs raised in an integrated swine production system. J Am Vet Med Assoc.

[pbio-0030015-b11] Engvall EO, Brandstrom B, Andersson L, Baverud V, Trowald-Wigh G (2003). Isolation and identification of thermophilic *Campylobacter* species in faecal samples from Swedish dogs. Scand J Infect Dis.

[pbio-0030015-b12] Hald B, Madsen M (1997). Healthy puppies and kittens as carriers of *Campylobacter* spp., with special reference to Campylobacter upsaliensis. J Clin Microbiol.

[pbio-0030015-b13] Moser I, Rieksneuwohner B, Lentzsch P, Schwerk P, Wieler LH (2001). Genomic heterogeneity and O-antigenic diversity of Campylobacter upsaliensis and Campylobacter helveticus strains isolated from dogs and cats in Germany. J Clin Microbiol.

[pbio-0030015-b14] Sandstedt K, Ursing J, Walder M (1983). Thermotolerant *Campylobacter* with no or weak catalase activity isolated from dogs. Curr Microbiol.

[pbio-0030015-b15] Shen Z, Feng Y, Dewhirst FE, Fox JG (2001). Coinfection of enteric *Helicobacter* spp. and *Campylobacter* spp. in cats. J Clin Microbiol.

[pbio-0030015-b16] Tresierra-Ayala A, Bendayan ME, Bernuy A, Pereyra G, Fernandez H (1994). Chicken as potential contamination source of Campylobacter lari in Iquitos, Peru. Rev Inst Med Trop Sao Paulo.

[pbio-0030015-b17] Moore JE, Madden RH (1998). Occurrence of thermophilic *Campylobacter* spp. in porcine liver in Northern Ireland. J Food Prot.

[pbio-0030015-b18] Kramer JM, Frost JA, Bolton FJ, Wareing DR (2000). *Campylobacter* contamination of raw meat and poultry at retail sale: Identification of multiple types and comparison with isolates from human infection. J Food Prot.

[pbio-0030015-b19] Park CE, Sanders GW (1992). Occurrence of thermotolerant campylobacters in fresh vegetables sold at farmers' outdoor markets and supermarkets. Can J Microbiol.

[pbio-0030015-b20] Endtz HP, Vliegenthart JS, Vandamme P, Weverink HW, van den Braak NP (1997). Genotypic diversity of Campylobacter lari isolated from mussels and oysters in the Netherlands. Int J Food Microbiol.

[pbio-0030015-b21] Rosef O, Rettedal G, Lageide L (2001). Thermophilic campylobacters in surface water: A potential risk of campylobacteriosis. Int J Environ Health Res.

[pbio-0030015-b22] Bourke B, Chan VL, Sherman P (1998). *Campylobacter upsaliensis* Waiting in the wings. Clin Microbiol Rev.

[pbio-0030015-b23] Atanassova V, Ring C (1999). Prevalence of *Campylobacter* spp. in poultry and poultry meat in Germany. Int J Food Microbiol.

[pbio-0030015-b24] Goossens H, Vlaes L, Butzler JP, Adnet A, Hanicq P (1991). Campylobacter upsaliensis enteritis associated with canine infections. Lancet.

[pbio-0030015-b25] Gurgan T, Diker KS (1994). Abortion associated with Campylobacter upsaliensis. J Clin Microbiol.

[pbio-0030015-b26] Walmsley SL, Karmali MA (1989). Direct isolation of atypical thermophilic *Campylobacter* species from human feces on selective agar medium. J Clin Microbiol.

[pbio-0030015-b27] Goossens H, Giesendorf BA, Vandamme P, Vlaes L, Van den Borre C (1995). Investigation of an outbreak of Campylobacter upsaliensis in day care centers in Brussels: Analysis of relationships among isolates by phenotypic and genotypic typing methods. J Infect Dis.

[pbio-0030015-b28] Parkhill J, Wren BW, Mungall K, Ketley JM, Churcher C (2000). The genome sequence of the food-borne pathogen Campylobacter jejuni reveals hypervariable sequences. Nature.

[pbio-0030015-b29] Gilbert M, Brisson JR, Karwaski MF, Michniewicz J, Cunningham AM (2000). Biosynthesis of ganglioside mimics in Campylobacter jejuni OH4384. Identification of the glycosyltransferase genes, enzymatic synthesis of model compounds, and characterization of nanomole amounts by 600-mhz (1)h and (13)c NMR analysis. J Biol Chem.

[pbio-0030015-b30] Ahmed IH, Manning G, Wassenaar TM, Cawthraw S, Newell DG (2002). Identification of genetic differences between two Campylobacter jejuni strains with different colonization potentials. Microbiology.

[pbio-0030015-b31] Miller WG, Bates AH, Horn ST, Brandl MT, Wachtel MR (2000). Detection on surfaces and in Caco-2 cells of Campylobacter jejuni cells transformed with new *gfp, yfp* and *cfp* marker plasmids. Appl Environ Microbiol.

[pbio-0030015-b32] Tauxe RV, Patton CM, Edmonds P, Barrett TJ, Brenner DJ (1985). Illness associated with *Campylobacter laridis* a newly recognized *Campylobacter* species. J Clin Microbiol.

[pbio-0030015-b33] le Roux E, Lastovica AJ, Lastovica AJ, Newell DG, Lastovica EE (1998). The Cape Town Protocol: How to isolate the most campylobacters for your dollar, pound, franc, yen, etc. Campylobacter, Helicobacter and related organisms.

[pbio-0030015-b34] Dingle KE, Colles FM, Ure R, Wagenaar JA, Duim B (2002). Molecular characterization of Campylobacter jejuni clones: A basis for epidemiological investigation. Emerg Infect Dis.

[pbio-0030015-b35] Eisen JA, Heidelberg JF, White O, Salzberg SL (2000). Evidence for symmetric chromosomal inversions around the replication origin in bacteria. Genome Biol.

[pbio-0030015-b36] Brown JR, Douady CJ, Italia MJ, Marshall WE, Stanhope MJ (2001). Universal trees based on large combined protein sequence data sets. Nat Genet.

[pbio-0030015-b37] Santos SR, Ochman H (2004). Identification and phylogenetic sorting of bacterial lineages with universally conserved genes and proteins. Environ Microbiol.

[pbio-0030015-b38] Nelson KE, Fouts DE, Mongodin EF, Ravel J, DeBoy RT (2004). Whole genome comparisons of serotype 4b and 1/2a strains of the food-borne pathogen Listeria monocytogenes reveal new insights into the core genome components of this species. Nucleic Acids Res.

[pbio-0030015-b39] Kuroda M, Ohta T, Uchiyama I, Baba T, Yuzawa H (2001). Whole genome sequencing of meticillin-resistant Staphylococcus aureus. Lancet.

[pbio-0030015-b40] Baba T, Takeuchi F, Kuroda M, Yuzawa H, Aoki K (2002). Genome and virulence determinants of high virulence community-acquired MRSA. Lancet.

[pbio-0030015-b41] Beres SB, Sylva GL, Barbian KD, Lei B, Hoff JS (2002). Genome sequence of a serotype M3 strain of group A *Streptococcus*: Phage-encoded toxins, the high-virulence phenotype, and clone emergence. Proc Natl Acad Sci U S A.

[pbio-0030015-b42] Perna NT, Plunkett G, Burland V, Mau B, Glasner JD (2001). Genome sequence of enterohaemorrhagic Escherichia coli O157:H7. Nature.

[pbio-0030015-b43] Morgan GJ, Hatfull GF, Casjens S, Hendrix RW (2002). Bacteriophage Mu genome sequence: Analysis and comparison with Mu-like prophages in *Haemophilus*, *Neisseria* and *Deinococcus*. J Mol Biol.

[pbio-0030015-b44] Suerbaum S, Josenhans C, Sterzenbach T, Drescher B, Brandt P (2003). The complete genome sequence of the carcinogenic bacterium Helicobacter hepaticus. Proc Natl Acad Sci U S A.

[pbio-0030015-b45] Hendrix RW, Lawrence JG, Hatfull GF, Casjens S (2000). The origins and ongoing evolution of viruses. Trends Microbiol.

[pbio-0030015-b46] Desiere F, McShan WM, van Sinderen D, Ferretti JJ, Brüssow H (2001). Comparative genomics reveals close genetic relationships between phages from dairy bacteria and pathogenic *Streptococci* Evolutionary implications for prophage–host interactions. Virology.

[pbio-0030015-b47] Wagner PL, Waldor MK (2002). Bacteriophage control of bacterial virulence. Infect Immun.

[pbio-0030015-b48] Bacon DJ, Alm RA, Hu L, Hickey TE, Ewing CP (2002). DNA sequence and mutational analyses of the pVir plasmid of Campylobacter jejuni 81–176. Infect Immun.

[pbio-0030015-b49] Cascales E, Christie PJ (2003). The versatile bacterial type IV secretion systems. Nat Rev Microbiol.

[pbio-0030015-b50] Ding Z, Atmakuri K, Christie PJ (2003). The outs and ins of bacterial type IV secretion substrates. Trends Microbiol.

[pbio-0030015-b51] Cascales E, Christie PJ (2004). Definition of a bacterial type IV secretion pathway for a DNA substrate. Science.

[pbio-0030015-b52] Schouls LM, Reulen S, Duim B, Wagenaar JA, Willems RJ (2003). Comparative genotyping of Campylobacter jejuni by amplified fragment length polymorphism, multilocus sequence typing, and short repeat sequencing: Strain diversity, host range, and recombination. J Clin Microbiol.

[pbio-0030015-b53] Miller WG, Keech AM, Pearson BM, Wells JM, Kapitonov VV (2005). Diversity of Campylobacter jejuni Type I restriction-modification loci: Induction of *hsdS* by exogenous DNA. Microbiology.

[pbio-0030015-b54] Kobayashi I (2001). Behavior of restriction-modification systems as selfish mobile elements and their impact on genome evolution. Nucleic Acids Res.

[pbio-0030015-b55] Wang Y, Taylor DE (1990). Natural transformation in *Campylobacter* species. J Bacteriol.

[pbio-0030015-b56] Suerbaum S, Lohrengel M, Sonnevend A, Ruberg F, Kist M (2001). Allelic diversity and recombination in Campylobacter jejuni. J Bacteriol.

[pbio-0030015-b57] Wassenaar TM, Fry BN, van der Zeijst BA (1993). Genetic manipulation of *Campylobacter*: Evaluation of natural transformation and electro-transformation. Gene.

[pbio-0030015-b58] Carlone GM, Lascelles J (1982). Aerobic and anaerobic respiratory systems in Campylobacter fetus subsp. *jejuni* grown in atmospheres containing hydrogen. J Bacteriol.

[pbio-0030015-b59] Smibert RM, Holt JG, Krieg NR (1984). *Campylobacter*. Bergey's manual of systematic bacteriology.

[pbio-0030015-b60] Mohammed KA, Miles RJ, Halablab MA (2004). The pattern and kinetics of substrate metabolism of Campylobacter jejuni and Campylobacter coli. Lett Appl Microbiol.

[pbio-0030015-b61] Konkel ME, Klena JD, Rivera-Amill V, Monteville MR, Biswas D (2004). Secretion of virulence proteins from Campylobacter jejuni is dependent on a functional flagellar export apparatus. J Bacteriol.

[pbio-0030015-b62] Wiesner RS, Hendrixson DR, DiRita VJ (2003). Natural transformation of Campylobacter jejuni requires components of a type II secretion system. J Bacteriol.

[pbio-0030015-b63] Jacob-Dubuisson F, Locht C, Antoine R (2001). Two-partner secretion in Gram-negative bacteria: A thrifty, specific pathway for large virulence proteins. Mol Microbiol.

[pbio-0030015-b64] Ketley JM (1997). Pathogenesis of enteric infection by *Campylobacter*. Microbiology.

[pbio-0030015-b65] Wassenaar TM, van der Zeijst BA, Ayling R, Newell DG (1993). Colonization of chicks by motility mutants of Campylobacter jejuni demonstrates the importance of flagellin A expression. J Gen Microbiol.

[pbio-0030015-b66] Yao R, Burr DH, Doig P, Trust TJ, Niu H (1994). Isolation of motile and non-motile insertional mutants of *Campylobacter jejuni* The role of motility in adherence and invasion of eukaryotic cells. Mol Microbiol.

[pbio-0030015-b67] Brás AM, Chatterjee S, Wren BW, Newell DG, Ketley JM (1999). A novel Campylobacter jejuni two-component regulatory system important for temperature-dependent growth and colonization. J Bacteriol.

[pbio-0030015-b68] Konkel ME, Garvis SG, Tipton SL, Anderson DE, Cieplak W (1997). Identification and molecular cloning of a gene encoding a fibronectin-binding protein (CadF) from Campylobacter jejuni. Mol Microbiol.

[pbio-0030015-b69] McCormick BA, Nusrat A, Parkos CA, D'Andrea L, Hofman PM (1997). Unmasking of intestinal epithelial lateral membrane beta1 integrin consequent to transepithelial neutrophil migration in vitro facilitates *inv*-mediated invasion by Yersinia pseudotuberculosis. Infect Immun.

[pbio-0030015-b70] Monteville MR, Konkel ME (2002). Fibronectin-facilitated invasion of T84 eukaryotic cells by Campylobacter jejuni occurs preferentially at the basolateral cell surface. Infect Immun.

[pbio-0030015-b71] Muza-Moons MM, Koutsouris A, Hecht G (2003). Disruption of cell polarity by enteropathogenic Escherichia coli enables basolateral membrane proteins to migrate apically and to potentiate physiological consequences. Infect Immun.

[pbio-0030015-b72] Isberg RR, Voorhis DL, Falkow S (1987). Identification of invasin: A protein that allows enteric bacteria to penetrate cultured mammalian cells. Cell.

[pbio-0030015-b73] Weiser JN, Lindberg AA, Manning EJ, Hansen EJ, Moxon ER (1989). Identification of a chromosomal locus for expression of lipopolysaccharide epitopes in Haemophilus influenzae. Infect Immun.

[pbio-0030015-b74] Serino L, Virji M (2002). Genetic and functional analysis of the phosphorylcholine moiety of commensal *Neisseria* lipopolysaccharide. Mol Microbiol.

[pbio-0030015-b75] Zhang JR, Idanpaan-Heikkila I, Fischer W, Tuomanen EI (1999). Pneumococcal *licD2* gene is involved in phosphorylcholine metabolism. Mol Microbiol.

[pbio-0030015-b76] Weiser JN, Shchepetov M, Chong ST (1997). Decoration of lipopolysaccharide with phosphorylcholine: A phase-variable characteristic of Haemophilus influenzae. Infect Immun.

[pbio-0030015-b77] Jennings MP, Srikhanta YN, Moxon ER, Kramer M, Poolman JT (1999). The genetic basis of the phase variation repertoire of lipopolysaccharide immunotypes in Neisseria meningitidis. Microbiology.

[pbio-0030015-b78] Hood DW, Deadman ME, Jennings MP, Bisercic M, Fleischmann RD (1996). DNA repeats identify novel virulence genes in Haemophilus influenzae. Proc Natl Acad Sci U S A.

[pbio-0030015-b79] Moran AP, Appelmelk BJ, Aspinall GO (1996). Molecular mimicry of host structures by lipopolysaccharides of *Campylobacter* and *Helicobacter* spp: Implications in pathogenesis. J Endotoxin Res.

[pbio-0030015-b80] Guerry P, Ewing CP, Hickey TE, Prendergast MM, Moran AP (2000). Sialylation of lipooligosaccharide cores affects immunogenicity and serum resistance of Campylobacter jejuni. Infect Immun.

[pbio-0030015-b81] Bacon DJ, Szymanski CM, Burr DH, Silver RP, Alm RA (2001). A phase-variable capsule is involved in virulence of Campylobacter jejuni 81–176. Mol Microbiol.

[pbio-0030015-b82] Gilbert M, Godschalk PCR, Parker CT, Endtz HP, Wakarchuk WW, Ketley J, Konkel ME (2004). Genetic basis for the variation in the lipooligosaccharide outer core of Campylobacter jejuni and possible association of glycosyltransferase genes with post-infectious neurophathies. *Campylobacter jejuni*: New perspectives in molecular and cellular biology.

[pbio-0030015-b83] Piddock LJ, Ricci V, Pumbwe L, Everett MJ, Griggs DJ (2003). Fluoroquinolone resistance in *Campylobacter* species from man and animals: Detection of mutations in topoisomerase genes. J Antimicrob Chemother.

[pbio-0030015-b84] Taylor DE, Ge Z, Purych D, Lo T, Hiratsuka K (1997). Cloning and sequence analysis of two copies of a 23S rRNA gene from Helicobacter pylori and association of clarithromycin resistance with 23S rRNA mutations. Antimicrob Agents Chemother.

[pbio-0030015-b85] Chopra I, Roberts M (2001). Tetracycline antibiotics: Mode of action, applications, molecular biology, and epidemiology of bacterial resistance. Microbiol Mol Biol Rev.

[pbio-0030015-b86] Trieber CA, Taylor DE (2002). Mutations in the 16S rRNA genes of Helicobacter pylori mediate resistance to tetracycline. J Bacteriol.

[pbio-0030015-b87] Gross CH, Parsons JD, Grossman TH, Charifson PS, Bellon S (2003). Active-site residues of Escherichia coli DNA gyrase required in coupling ATP hydrolysis to DNA supercoiling and amino acid substitutions leading to novobiocin resistance. Antimicrob Agents Chemother.

[pbio-0030015-b88] Gibreel A, Skold O (1999). Sulfonamide resistance in clinical isolates of *Campylobacter jejuni* Mutational changes in the chromosomal dihydropteroate synthase. Antimicrob Agents Chemother.

[pbio-0030015-b89] Heep M, Beck D, Bayerdorffer E, Lehn N (1999). Rifampin and rifabutin resistance mechanism in Helicobacter pylori. Antimicrob Agents Chemother.

[pbio-0030015-b90] Sutton GG, White O, Adams MD, Kerlavage AR (1995). TIGR Assembler: A new tool for assembling large shotgun sequencing projects. Genome Sequencing Tech.

[pbio-0030015-b91] Myers EW, Sutton GG, Delcher AL, Dew IM, Fasulo DP (2000). A whole-genome assembly of *Drosophila*. Science.

[pbio-0030015-b92] Biery MC, Stewart FJ, Stellwagen AE, Raleigh EA, Craig NL (2000). A simple in vitro Tn7-based transposition system with low target site selectivity for genome and gene analysis. Nucleic Acids Res.

[pbio-0030015-b93] Delcher AL, Phillippy A, Carlton J, Salzberg SL (2002). Fast algorithms for large-scale genome alignment and comparison. Nucleic Acids Res.

[pbio-0030015-b94] Pop M, Kosack DS, Salzberg SL (2004). Hierarchical scaffolding with Bambus. Genome Res.

[pbio-0030015-b95] Gajer P, Schatz M, Salzberg SL (2004). Automated correction of genome sequence errors. Nucleic Acids Res.

[pbio-0030015-b96] Delcher AL, Harmon D, Kasif S, White O, Salzberg SL (1999). Improved microbial gene identification with GLIMMER. Nucleic Acids Res.

[pbio-0030015-b97] Altschul SF, Gish W, Miller W, Myers EW, Lipman DJ (1990). Basic local alignment search tool. J Mol Biol.

[pbio-0030015-b98] Tomb JF, White O, Kerlavage AR, Clayton RA, Sutton GG (1997). The complete genome sequence of the gastric pathogen Helicobacter pylori. Nature.

[pbio-0030015-b99] Alm RA, Ling LS, Moir DT, King BL, Brown ED (1999). Genomic-sequence comparison of two unrelated isolates of the human gastric pathogen Helicobacter pylori. Nature.

[pbio-0030015-b100] Baar C, Eppinger M, Raddatz G, Simon J, Lanz C (2003). Complete genome sequence and analysis of Wolinella succinogenes. Proc Natl Acad Sci U S A.

[pbio-0030015-b101] Dingle KE, Colles FM, Wareing DR, Ure R, Fox AJ (2001). Multilocus sequence typing system for Campylobacter jejuni. J Clin Microbiol.

[pbio-0030015-b102] Meinersmann RJ, Helsel LO, Fields PI, Hiett KL (1997). Discrimination of Campylobacter jejuni isolates by *fla* gene sequencing. J Clin Microbiol.

[pbio-0030015-b103] Felsenstein J (2004). PHYLIP, version 3.6 [computer progam]. Seattle: Department of Genome Sciences, University of Washington, Seattle. Available: http://evolution.genetics.washington.edu/phylip.html. http://evolution.genetics.washington.edu/phylip.html.

[pbio-0030015-b104] Heidelberg JF, Seshadri R, Haveman SA, Hemme CL, Paulsen IT (2004). The genome sequence of the anaerobic, sulfate-reducing bacterium Desulfovibrio vulgaris Hildenborough. Nat Biotechnol.

[pbio-0030015-b105] Thompson JD, Higgins DG, Gibson TJ (1994). CLUSTALW: Improving the sensitivity of progressive multiple sequence alignment through sequence weighting, position-specific gap penalties and weight matrix choice. Nucleic Acids Res.

[pbio-0030015-b106] Ewing B, Hillier L, Wendl MC, Green P (1998). Base-calling of automated sequencer traces using phred. I. Accuracy assessment. Genome Res.

