# Supplementary material for: Major Structural Differences and Novel Potential Virulence Mechanisms from the Genomes of Multiple Campylobacter Species
Source: PLoS Biol. 2005 Jan 4;3(1):e15. doi: 10.1371/journal.pbio.0030015 (PMC539331; doi:10.1371/journal.pbio.0030015)
Supplement: Figure S2 — (274 KB PDF). [file pbio.0030015.sg002.pdf]

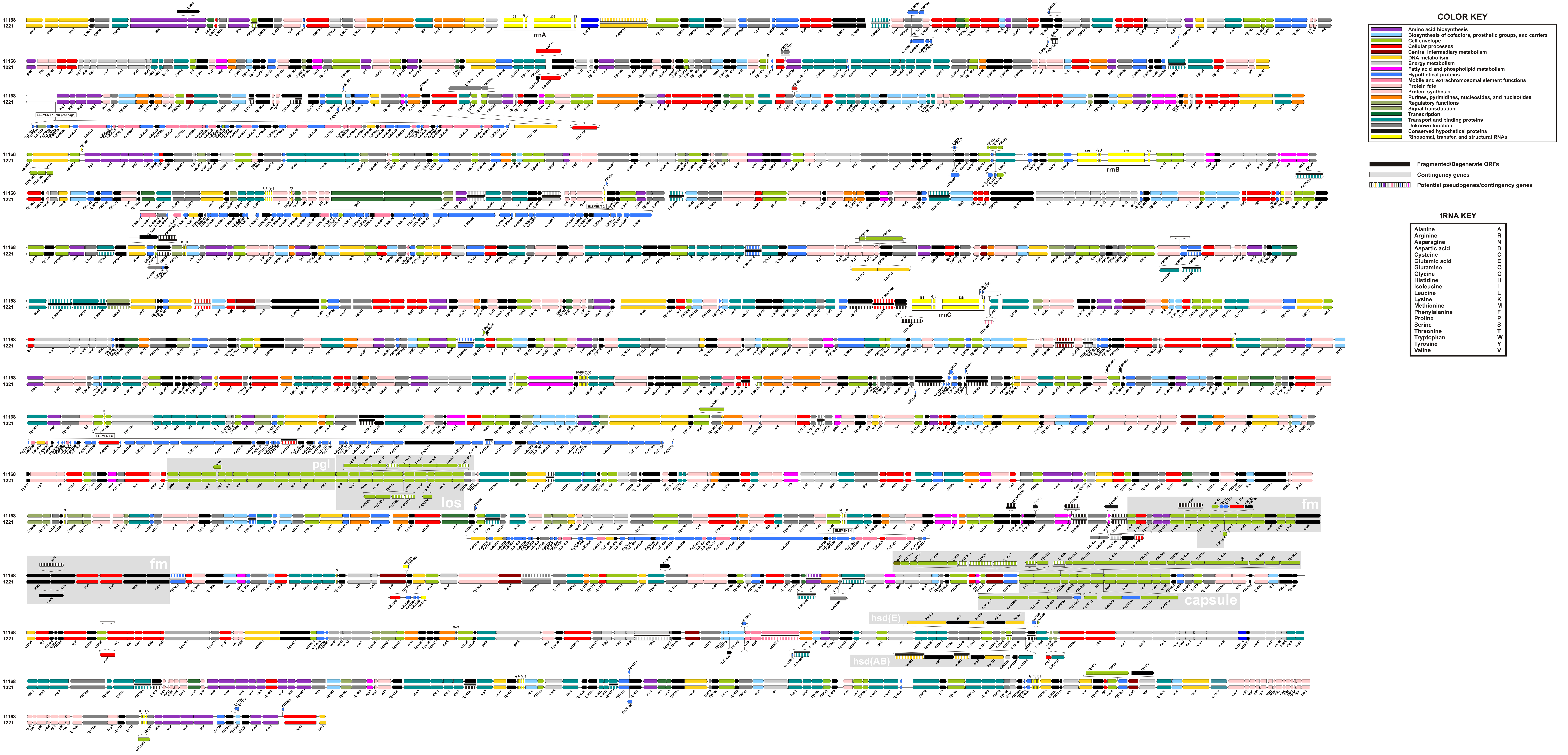
COLOR KEY
Amino acid biosynthesis
Biosynthesis of cofactors, prosthetic groups, and carriers
Cell envelope
Cellular processes
Central intermediary metabolism
DNA metabolism
Energy metabolism
Fatty acid and phospholipid metabolism
Hypothetical proteins
Mobile and extrachromosomal element functions
Protein fate
Protein synthesis
Purines, pyrimidines, nucleosides, and nucleotides
Regulatory functions
Signal transduction
Transcription
Transport and binding proteins
Unknown function
Conserved hypothetical proteins
Ribosomal, transfer, and structural RNAs
Fragmented/Degenerate ORFs
Contingency genes
Potential pseudogenes/contingency genes
tRNA KEY
Alanine A
Arginine R
Asparagine N
Aspartic acid D
Cysteine C
Glutamic acid E
Glutamine Q
Glycine G
Histidine H
Isoleucine I
Leucine L
Lysine K
Methionine M
Phenylalanine F
Proline P
Serine S
Threonine T
Tryptophan W
Tyrosine Y
Valine V
11168
1221
rrnA
rrnB
rrnC
ELEMENT 1 (mu prophage)
ELEMENT 2
ELEMENT 3
ELEMENT 4
pgl
los
fm
capsule
hsd(E)
hsd(AB)
