# Supplementary figures and images for: Major Structural Differences and Novel Potential Virulence Mechanisms from the Genomes of Multiple Campylobacter Species
Source: PLoS Biol. 2005 Jan 4;3(1):e15. doi: 10.1371/journal.pbio.0030015 (PMC539331; doi:10.1371/journal.pbio.0030015)

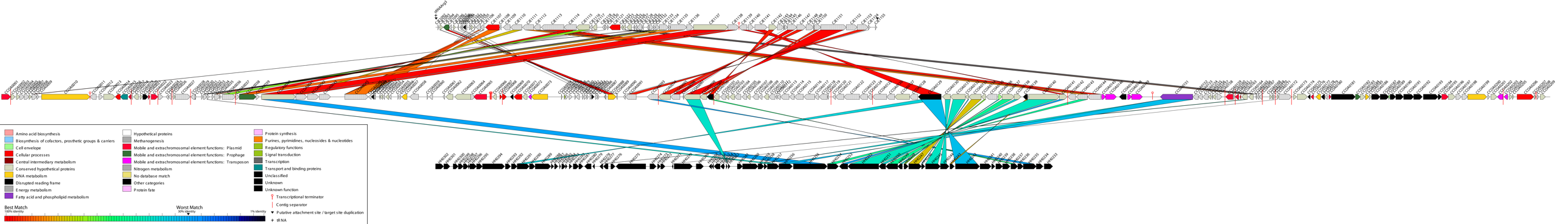

Supplement: Figure S3 — CJIE3 (top linear figure) and H. hepaticus ATCC 51449 HHGI1 (bottom line) against pCC178 megaplasmid of C. coli RM2228 (middle line). Colors of ORFs are indicated in the key by putative function. Connecting lines represent those ORFs whose protein sequences match at a BLASTP of 30% identity or better. These lines do not indicate the coordinates of match, merely that there is a match. (76 KB PDF). [file pbio.0030015.sg003.pdf]
