# Supplementary material for: Major Structural Differences and Novel Potential Virulence Mechanisms from the Genomes of Multiple Campylobacter Species
Source: PLoS Biol. 2005 Jan 4;3(1):e15. doi: 10.1371/journal.pbio.0030015 (PMC539331; doi:10.1371/journal.pbio.0030015)
Supplement: Figure S5 — The characters in italics indicate the 32-bp spacer sequences that are unique to the two strains; the spacer sequences for NCTC 11168 are 1 bp longer than presented by others [52]. The bold characters represent the CRISPR repeat region in RM1221 (n = 4) and NCTC 11168 (n = 5). The characters in roman typeface indicate regions flanking the repeat region that are identical in the two strains. (20 KB DOC). [file pbio.0030015.sg005.doc]

***C. jejuni* RM1221**

TAGTGGAGTAATTAGCCCTAGCGGAGTTTCAATCCACTAGGGTTTA

**TTTTAGTCCCTTTTTAAATTTCTTTATGGTAAAA** *TTCCATTCTCATGAAATATTTAGCCATTATTG*

**TTTTAGTCCCTTTTTAAATTTCTTTATGGTAAAA** *TGGTAGTAGAATTATCTTTGCTGGTATTAATG*

**TTTTAGTCCCTTTTTAAATTTCTTTATGGTAAAA** *TTCTAAAATTGTTTCATTTTCATTAGTAGCTG*

**TTTTAGTCCCTTTTTAAATTTCTTTATGGTAAAA** TAGATATTTACCAGATAATGAAAATTTCGGGG

***C. jejuni* NCTC 11168**

TAGTGGAGTAATTAGCCCTAGTGGAGTTTCAATCCACTAGGGTTTA

**TTTTAGTCCCTTTTTAAATTTCTTTATGGTAAAA** *TTATGGCAGTTTTTAAAAGAGCTTGGCGGTTG*

**TTTTAGTCCCTTTTTAAATTTCTTTATGGTAAAA** *TTTTCCAAAGTTTCATTAGTTGAATTTAACTG*

**TTTTAGTCCCTTTTTAAATTTCTTTATGGTAAAA** *TCTACAAGAATGAGGATGATGATATTTTACAG*

**TTTTAGTCCCTTTTTAAATTTCTTTATGGTAAAA** *TATGAGTGTGCTAAAAAAAATGGACTTAAATG*

**TTTTAGTCCCTTTTTAAATTTCTTTATGGTAAAA** TAGATATTTACCAGATAATGAAAATTTCGGGG
