# Supplementary material for: Major Structural Differences and Novel Potential Virulence Mechanisms from the Genomes of Multiple Campylobacter Species
Source: PLoS Biol. 2005 Jan 4;3(1):e15. doi: 10.1371/journal.pbio.0030015 (PMC539331; doi:10.1371/journal.pbio.0030015)
Supplement: Figure S6 — The tricarboxylic (TCA) cycle has major variations based on comparative analysis across the strains (please refer to text). Differences in substrate respiration based on an analysis of Biolog data and species-specific pathways are also presented in the text. (51 KB PPT). [file pbio.0030015.sg006.ppt]

## Slide 1
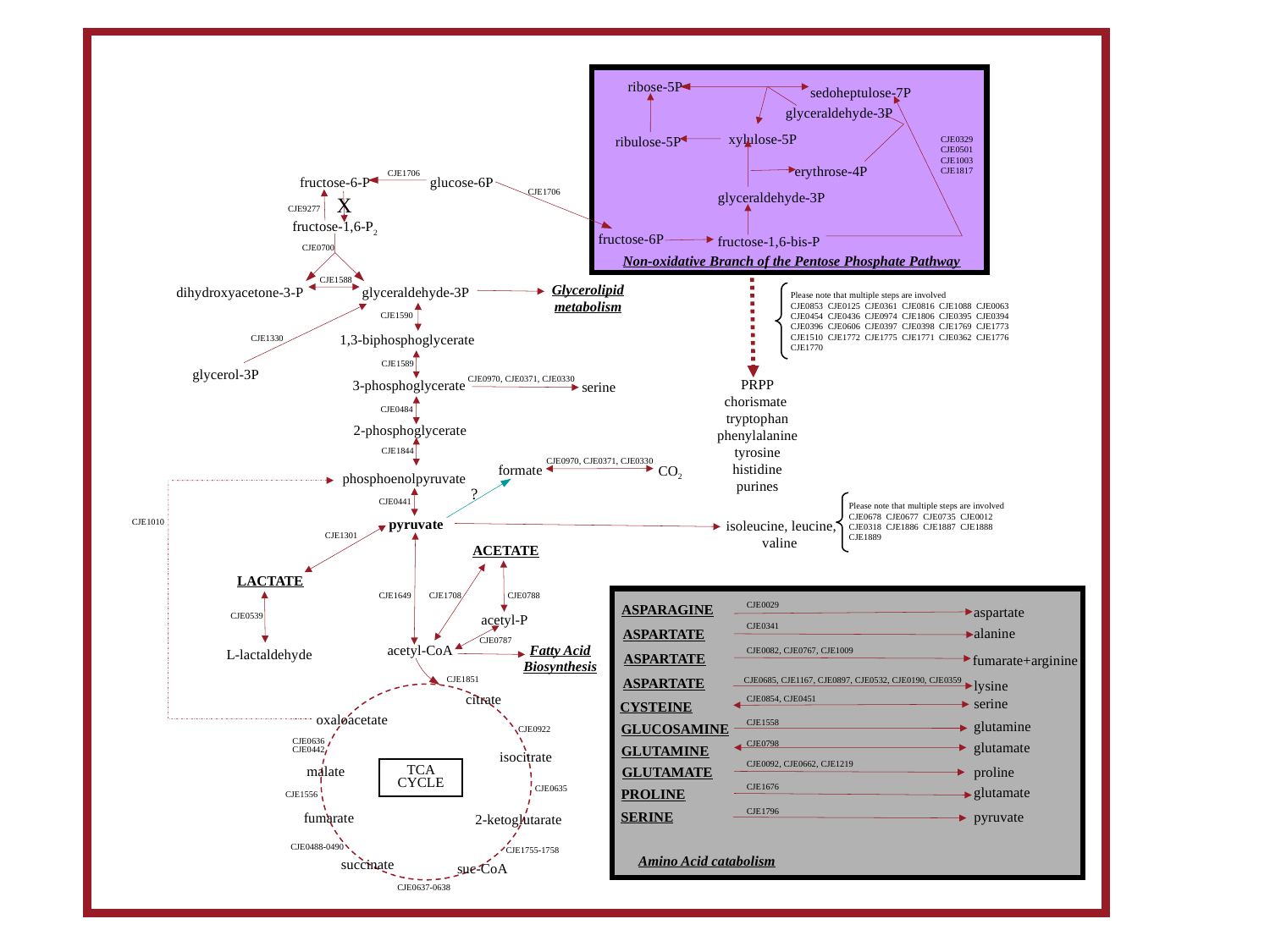

ribose-5P
sedoheptulose-7P
glyceraldehyde-3P
xylulose-5P
ribulose-5P
CJE0329
CJE0501
CJE1003
CJE1817
erythrose-4P
CJE1706
glucose-6P
fructose-6-P
CJE1706
glyceraldehyde-3P
X
CJE9277
fructose-1,6-P2
fructose-6P
fructose-1,6-bis-P
CJE0700
Non-oxidative Branch of the Pentose Phosphate Pathway
CJE1588
Glycerolipid metabolism
dihydroxyacetone-3-P
glyceraldehyde-3P
Please note that multiple steps are involved
CJE0853 CJE0125 CJE0361 CJE0816 CJE1088 CJE0063
CJE0454 CJE0436 CJE0974 CJE1806 CJE0395 CJE0394
CJE0396 CJE0606 CJE0397 CJE0398 CJE1769 CJE1773
CJE1510 CJE1772 CJE1775 CJE1771 CJE0362 CJE1776
CJE1770
CJE1590
1,3-biphosphoglycerate
CJE1330
CJE1589
glycerol-3P
CJE0970, CJE0371, CJE0330
PRPP
chorismate
tryptophan
phenylalanine
tyrosine
histidine
purines
3-phosphoglycerate
serine
CJE0484
2-phosphoglycerate
CJE1844
CJE0970, CJE0371, CJE0330
formate
CO2
phosphoenolpyruvate
?
CJE0441
Please note that multiple steps are involved
CJE0678 CJE0677 CJE0735 CJE0012
CJE0318 CJE1886 CJE1887 CJE1888
CJE1889
pyruvate
CJE1010
isoleucine, leucine,
valine
CJE1301
ACETATE
LACTATE
CJE1649
CJE1708
CJE0788
CJE0029
ASPARAGINE
aspartate
acetyl-P
CJE0539
CJE0341
alanine
ASPARTATE
CJE0787
Fatty Acid Biosynthesis
acetyl-CoA
CJE0082, CJE0767, CJE1009
L-lactaldehyde
ASPARTATE
fumarate+arginine
CJE1851
ASPARTATE
CJE0685, CJE1167, CJE0897, CJE0532, CJE0190, CJE0359
lysine
citrate
CJE0854, CJE0451
serine
CYSTEINE
oxaloacetate
glutamine
CJE1558
GLUCOSAMINE
CJE0922
CJE0636
CJE0442
glutamate
CJE0798
GLUTAMINE
isocitrate
CJE0092, CJE0662, CJE1219
malate
GLUTAMATE
proline
TCA CYCLE
CJE1676
glutamate
CJE0635
PROLINE
CJE1556
CJE1796
SERINE
pyruvate
fumarate
2-ketoglutarate
CJE0488-0490
CJE1755-1758
Amino Acid catabolism
succinate
suc-CoA
CJE0637-0638
